# Combining Multiple Hypothesis Testing with Machine Learning Increases the Statistical Power of Genome-wide Association Studies

**DOI:** 10.1038/srep36671

**Published:** 2016-11-28

**Authors:** Bettina Mieth, Marius Kloft, Juan Antonio Rodríguez, Sören Sonnenburg, Robin Vobruba, Carlos Morcillo-Suárez, Xavier Farré, Urko M. Marigorta, Ernst Fehr, Thorsten Dickhaus, Gilles Blanchard, Daniel Schunk, Arcadi Navarro, Klaus-Robert Müller

**Affiliations:** 1 Machine Learning Group, Technische Universität Berlin, Berlin, 10587, Germany; 2 Department of Computer Science, Humboldt University of Berlin, Berlin, 10099, Germany; 3 Institut de Biología Evolutiva (CSIC-UPF). Departament de Ciències Experimentals i de la Salut. Universitat Pompeu Fabra, Barcelona, 08003, Spain; 4 TomTom Research, Berlin, 12555, Germany; 5 School of Biology, Georgia Institute of Technology, Atlanta, 30332, GA, USA; 6 Department of Economics, Laboratory for Social and Neural Systems Research, University of Zurich, Zurich, 8006, Switzerland; 7 Institute for Statistics (FB 3), University of Bremen, Bremen, 28359, Germany; 8 Department of Mathematics, University of Potsdam, Potsdam, 14476, Germany; 9 Department of Economics, University of Mainz, Mainz, 55099, Germany; 10 Institució Catalana de Recerca i Estudis Avançats (ICREA), Barcelona, 08010, Spain; 11 Center for Genomic Regulation (CRG), Barcelona Institute of Science and Technology (BIST), Barcelona, 08003, Spain; 12 Department of Brain and Cognitive Engineering, Korea University, Seoul, Republic of Korea

## Abstract

The standard approach to the analysis of genome-wide association studies (GWAS) is based on testing each position in the genome individually for statistical significance of its association with the phenotype under investigation. To improve the analysis of GWAS, we propose a combination of machine learning and statistical testing that takes *correlation structures* within the set of SNPs under investigation in a mathematically well-controlled manner into account. The novel two-step algorithm, COMBI, first trains a support vector machine to determine a subset of candidate SNPs and then performs hypothesis tests for these SNPs together with an adequate threshold correction. Applying COMBI to data from a WTCCC study (2007) and measuring performance as replication by independent GWAS published within the 2008–2015 period, we show that our method outperforms ordinary raw *p*-value thresholding as well as other state-of-the-art methods. COMBI presents higher power and precision than the examined alternatives while yielding fewer *false* (*i.e*. non-replicated) and more *true* (*i.e*. replicated) discoveries when its results are validated on later GWAS studies. More than 80% of the discoveries made by COMBI upon WTCCC data have been validated by independent studies. Implementations of the COMBI method are available as a part of the GWASpi toolbox 2.0.

The goal of genome-wide association studies (GWAS) (e.g. the WTCCC study^1^) is to examine the relationship between genetic markers such as single-nucleotide polymorphisms (SNPs) and individual traits, which are usually complex diseases or behavioral characteristics. Generally, a large number of statistical tests are performed in parallel, each SNP being *individually* tested for association^2^,^3^,^4^. The standard approach consists of computing individual, SNP-specific *p*-values corresponding to a statistical association test and comparing these *p*-values against some given significance threshold (say t^*^), meaning that precisely those SNPs with *p*-values smaller than t^*^are declared to be associated with the trait^4^,^5^,^6^. We refer to this approach as raw *p*-value thresholding (RPVT) and review some standard methods for choosing t^*^for the purpose of controlling multiple type I error rates (in particular, the family-wise error rate (*FWER*) and the expected number of false rejections (*ENFR*)) in the Methods Section.

According to the GWAS catalog^7^,^8^ (last accessed 03-07-2015), the more than 1,400 GWAS published so far have led to the identification of more than 11,000 SNPs associated with about 800 human diseases and anthropometric traits with *p*-values using t^*^ = 1 × 10^−5^.

However, variants reported by GWAS tend to explain only small fractions of individual traits, and most of the heritability accounting for many complex diseases remains unexplained — a phenomenon usually referred to as the “mystery of missing heritability”^4^,^9^. There are several possible (not mutually exclusive) explanations for that phenomenon^10^,^11^,^12^,^13^. One frequently discussed possibility is that epistatic interactions between loci are ignored both in current heritability estimates and in usual testing procedures^12^,^14^. In addition to this issue, another shortcoming of current approaches based on testing each SNP independently is that they disregard any correlation structures among the set of SNPs under investigation that are introduced by both population genetics (linkage disequilibrium, LD) and biological relations (*e.g*. functional relationships between genes). The latter issue by itself is likely to introduce confounding factors and artifacts, implying a loss in statistical power^15^ and a lack of reliable insights about genotype-phenotype associations.

In this work, we propose a novel methodology — COMBI — that is a principled, reliable, and replicable method for identifying significant SNP-phenotype associations. The core idea is a two-step algorithm consisting of

1. a machine learning and SNP selection step that drastically reduces the number of candidate SNPs by selecting only a small subset of the most predictive SNPs; and
2. a statistical testing step where only the SNPs selected in step 1 are tested for association.

The main idea underlying COMBI is the use of the state-of-the-art machine learning technique support vector machine (SVM)^16^,^17^,^18^ in the first step. Crucially, this method is tailored to predict the target output (here, the phenotype) from high-dimensional data with a possibly complex, unknown correlation structure. In our application, the SVM is trained using the complete SNP data of one chromosome. Thus, the first step acts as a filter, indicating SNPs that are relevant for phenotype classification with either high individual effects or effects in combination with the rest of SNPs, while discarding artifacts due to the correlation structure. The second step uses multiple statistical hypotheses testing for a quantitative assessment of individual relevance of the filtered SNPs. All in all, the two steps extract complementary types of information, which are combined in the final output. Importantly, the calibration of the method is such that a global statistical error criterion is controlled for the entire procedure consisting of steps 1 and 2.

The following section first introduces the methodology in a summary paragraph and in Fig. 1; then, the Methods Section continues to explain the method in more detail with some references to Supplementary Section 1. An overview of related machine learning work is given in the Discussion Section. The performance of the COMBI method is reported in the Results Section, Supplementary Sections 2 and 3; where we also include and discuss the highly favorable comparisons with the algorithms that could potentially compete with the COMBI method. Note that COMBI yields better prediction with fewer *false* (*i.e*. non-replicated) and more *true* (*i.e*. replicated) discoveries when its results are validated on later, larger GWAS studies.

Implementations of the COMBI method are available in R, MATLAB, and JAVA, as a part of the GWASpi toolbox 2.0 (https://bitbucket.org/gwas_combi/gwaspi/).

## Methods

### Summary of The COMBI Method

**Figure 1:**
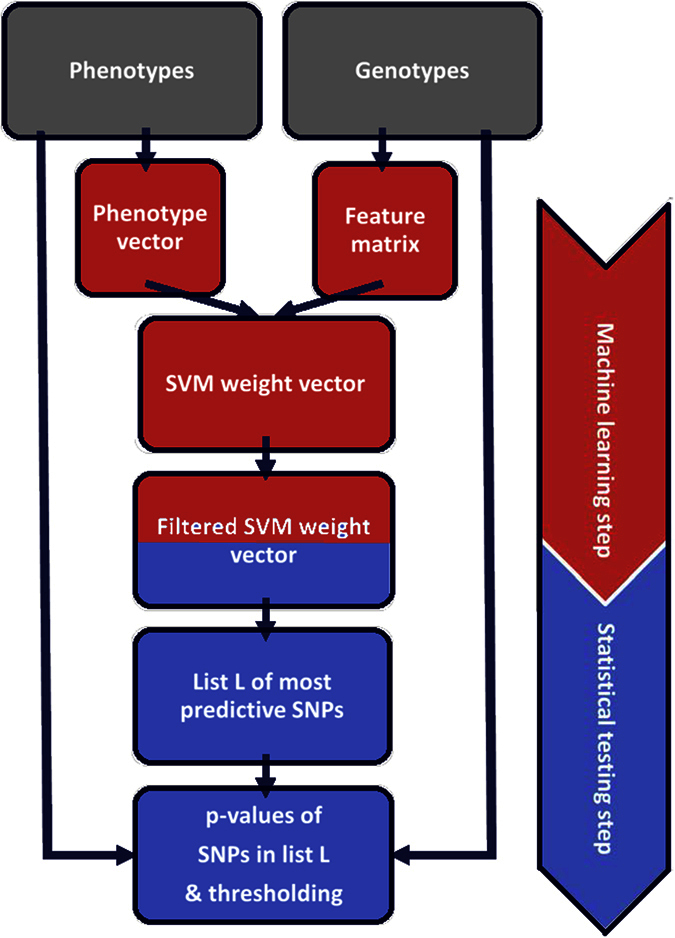
The COMBI method - Summary and illustration of the methodology. Receiving genotypes and corresponding phenotypes of a GWAS as input, the COMBI method first applies a machine learning step to select a set of candidate SNPs and then calculates *p*-values and corresponding significance thresholds in a statistical testing step.

Figure 1 shows a graphical representation of the COMBI method.

**Input:** a sample of observed genotypes {*x*_i*_} and corresponding phenotypes {*y*_*i*_}. We represent the *j*-th SNP of the *i*-th subject with a binary genotypic encoding, where *x*_*ij*_ = (1, 0, 0), *x*_*ij*_ = (0, 1, 0), or *x*_*ij*_ = (0, 0, 1), depending on the number of minor alleles. We assume a binary phenotype, i.e., *y*_*i*_ ∈ {+1, −1}.

- **Machine learning and SNP-selection step (colored in red).** Based on the sample, an SVM is trained. The SVM returns a linear function *f*(*x*) = *w*^*T*^*x*, the sign of *f*(*x*) is a prediction of the unknown phenotype of a previously unseen genotype *x*. The absolute value *|w*_*j*_*|* of the corresponding component of the parameter vector *w* is interpreted for each SNP j as a measure of importance for the prediction function. The parameter vector *w* is then post-processed through a *p*-th-order moving average filter with window size *l*, that is, 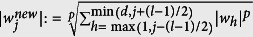. Finally, the SNPs corresponding to the *k* largest values of the scores are selected; all other SNPs are discarded.
- **Statistical testing step (colored in blue).** A hypothesis test (carried out as a *χ*^2^ test) is performed for each of the selected SNPs. Those SNPs with *p*-value less than a significance threshold t* are returned. The threshold t* is calibrated using a permutation-based method over the *whole* procedure consisting of the machine learning selection and statistical testing steps. See Algorithm 2 for details.

### Problem Setting and Methodology

In this section, we formally describe the statistical problem under investigation and propose a novel methodology for tackling it — based on a combination of machine learning and statistical testing techniques.

### Problem Setting and Notation

Let *n* denote the number of subjects in the study and *d* the number of SNPs under investigation. Given a sample of observed genotypes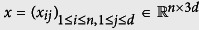 and corresponding phenotypes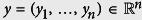, each *x*_i*_ and each x_*j_ corresponds to a subject and a SNP, respectively. A binary feature encoding is employed, where *x*_*ij*_ = (1, 0, 0), *x*_*ij*_ = (0, 1, 0), or *x*_*ij*_ = (0, 0, 1) depending on the number of minor alleles in SNP *j* of subject *i*. This paper focuses on binary phenotypes, i.e., *y*_*i*_ ∈ {+1, −1} for all *i* = l, …, *n*. The data for one particular SNP can be summarized in a contingency table (See Table 1).

The numbers n_ik_ denote the number of cases (*i* = 1) and controls (*i* = 2), respectively, which exhibit the genotype corresponding to column *k*. Notice that the row sums *n*_1._ and *n*_2._ are fixed and non-random by the experimental design (case-control study). Hence, the random vectors (*n*_11_, *n*_12_, *n*_13_)^T^ and (*n*_21_, *n*_22_, *n*_23_)^T^ follow a multinomial distribution with three categories, sample sizes *n*_1._ and *n*_2._, respectively; and unknown vectors of probabilities *p*_1_ = (*p*_11_, *p*_12_, *p*_13_)^T^ and *p*_2_ = (*p*_21_, *p*_22_, *p*_23_)^T^, respectively. The parameter 
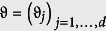
 of the statistical model for the whole study thus consists of all such pairs 
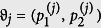
 of multinomial probability vectors, one for each of the d SNPs under investigation. For every SNP *j*, we are interested in testing the null hypothesis 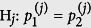, where we introduced the superscript *j* to indicate the SNP. This hypothesis is equivalent to the null hypothesis that the genotype at locus *j* is independent of the binary trait of interest. Two standard asymptotic tests for H_*j*_ versus its two-sided alternative K_*j*_ (genotype *j* is associated with the trait) are: the chi-square test for association and the Cochran-Armitage trend test (see, *e.g*., Sections 3.2.1 and 5.3.5 of the monograph by Agresti^19^). Both tests employ test statistics which are asymptotically (as min(*n*_1._, *n*_2._) tends to infinity) chi-square distributed under H_*j*_; the number of degrees of freedom equals 2 for the chi-square test for association, and 1 for the Cochran-Armitage trend test. Thus, *p*-values (*p*_*j*_: 1 ≤ *j* ≤ *d*) corresponding to these tests can be calculated by applying the upper-tail distribution function of the chi-square distribution with the corresponding degrees of freedom to the observed values of these statistics, and this for every SNP. Observe that the test statistics obtained for different SNPs will be highly correlated if these SNPs are in strong LD to each other; consequently, the corresponding *p*-values will also exhibit strong dependencies^20^,^21^.

RPVT declares a SNP *j* significantly associated with the trait if *p*_*j*_ ≤ t^*^. If there was a single test to perform (*i.e*., *d* = 1), then t^*^ would be taken as a pre-defined significance level α, as in the classical approach to statistical hypothesis testing. In multiple testing, however, the threshold t^*^ is modified to take the multiplicity of the problem (the fact that *d* > 1) into account. The simplest method is the so-called Bonferroni correction, 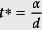. This choice guarantees that the *FWER* (that is, the probability of one or more erroneously reported associations) of the multiple test is bounded by α. A variety of other RPVT methods are explained, for instance, in the monograph by Dickhaus^22^.

### Proposed workflow

The Bonferroni correction can only attain the prescribed *FWER* upper bound, and therefore have maximal power, if the *p*-values (*p*_*j*_:1 ≤ *j* ≤ *d*) do not exhibit strong (positive) dependencies, an assumption which is violated in GWAS due to strong LD in blocks of SNPs. An alternative way to calibrate the threshold t^*^ for *FWER* control, taking the dependencies into account, is the Westfall-Young permutation procedure^23^, which controls the *FWER* under an assumption termed *subset pivotality* (see Westfall and Young^23^ as well as Dickhaus and Stange^21^). Furthermore, Meinshausen *et al*.^24^ proved that this permutation procedure is asymptotically optimal in the class of RPVT procedures, provided that the subset pivotality condition is fulfilled. However, for RPVT the individual *p*-value for association of the *j*-th SNP only depends on x_*j_ and thus ignores the possible correlations with the rest of the genotype – which could yield additional information. By contrast, machine learning approaches aimed at prediction try to take the information of the whole genotype into account at once, and thus implicitly consider all possible correlations, to strive for an optimal prediction of the phenotype. Based on this observation, we propose Algorithm 1 combining the advantages of the two techniques, consisting of the following two steps:

- the machine learning step, where an appropriate subset of candidate SNPs is selected, based on their relevance for prediction of the phenotype;
- the statistical testing step, where a hypothesis test is performed together with a Westfall-Young type threshold calibration for each SNP.

Additionally, a filter first processes the weight vector *w* output in the machine learning step before using it for the selection of candidate SNPs. The above steps are discussed in more detail in the following sections.

### The machine learning and SNP selection step

The goal in machine learning is to determine, based on the sample, a function *f*(*x*) that predicts the unknown phenotype *y* based on the observation of genotype *x*. It is crucial to require such a function to not only capture the sample at hand, but to also *generalize*, as well as possible, to new and unseen measurements, i.e., the sign of *f*(*x*) is a good predictor for *y* for previously unseen patterns *x* and labels *y*. We consider linear models of the form *f*_*w*,*b*_(*x*_i*_) = *w*^T^*x*_i*_ + *b* in this paper. A popular approach to learning such a model is given by the SVM^16^,^17^,^18^, which determines the parameter *w* of the model by solving, for *C* > *0*, the following optimization problem:


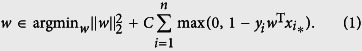


The problem above is similar to regression problems and can be interpreted as follows: we aim to minimize the trade-off (controlled by *C*) between a vector *w* with small norm (the term on the left-hand side) and small errors on the data (the term on the right-hand side). Once a classification function *f* has been determined by solving the above optimization problem, it can be used to predict the phenotype of any genotype by putting


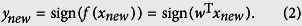


The above equation shows that the largest components (in absolute value) of the vector *w* (called SVM *parameter* or *weight* vector) also have the most influence on the predicted phenotype. Note that the weights vector contains three values for each position due to the feature embedding, which encodes each SNP with three binary variables. To convert the vector back to the original length, we simply take the average over the three weights. We also include an offset by including a constant feature that is all one.

Considering that the use of SVM weights as importance measures is a standard approach^25^, for each *j* the score *abs*(*w*_*j*_) can be interpreted as a measure for the importance of the *j*-th SNP for the phenotype prediction task. The main idea is to select only a small number *k* of candidate SNPs before statistical testing, namely those SNPs having the largest scores. Based on preliminary experiments, we noticed that the introduction of the following additional post-processing of the SVM parameter vector was beneficial before SNP selection: a *p*th-order moving average filter is applied as follows:


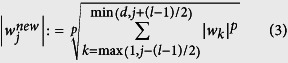


where *l* ∈ 1, …, *d* denotes a fixed filter length (required to be an odd number). The value *p* ∈ ]0, ∞[is a free parameter; in the case *p* = 1, a standard moving average filter is obtained.

### The statistical testing step

In the statistical testing step (see Summary of the COMBI method and Fig. 1), we apply *p*-value thresholding only to the *k p*-values which correspond to the SNPs with largest filtered SVM weights. Calculation of these *p*-values is performed exactly as described above for RPVT, with the only modification that *p*-values for SNPs not ranked among the top *k* in terms of their filtered SVM weights are set to 1, without calculating a test statistic.

The methodological challenge now consists of finding a threshold t^*^for the remaining *k p*-values such that the *FWER* is controlled for the multiple test which the entire workflow defines (SVM training, filtering of weights, *p*-value calculation, *p*-value thresholding). To this end, we investigated prior approaches^26^,^27^ based on sample splitting meaning that the selection of *k* SNPs is done on one (randomly chosen) sub-sample of individuals, while the *p*-value calculation and thresholding for the selected SNPs is performed on another. In this scheme, and regardless of which SNP selection method used on the first sub-sample, a Bonferroni-type threshold 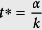 guarantees *FWER* control at level *α* for the *p*-values computed on the second sub-sample. Since *k* ≪ *d*, this correction is much less conservative than the original Bonferroni correction using all SNPs. However, this is severely mitigated by the loss of power in the *p*-values due to the sample splitting. In fact, computer simulations (See Supplementary Section 2.2.4.) indicated very low power for detecting true associations with such a method because of the reduced sample size for calculation of test statistics and *p*-values.

Our suggestion is to re-sample the entire workflow of Fig. 1, thus following a Westfall and Young^23^ type procedure, and to choose t* based on the permutation distribution of the re-sampled *p*-values.

In summary, the proposed methodology is formally stated as Algorithm 1.


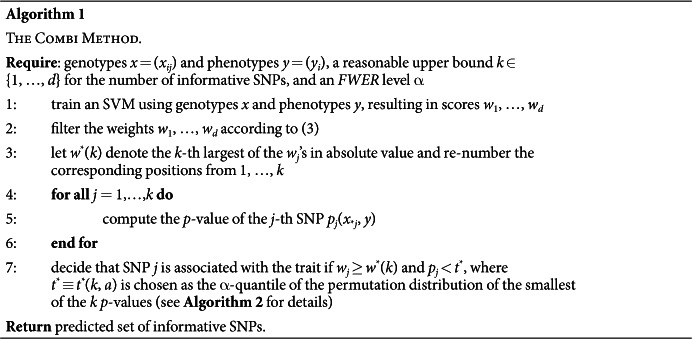


*FWER* control at level α of the multiple test defined by Algorithm 1 can be proven under a relaxed form of the subset pivotality condition, the validity of which is checked empirically in Supplementary Sections 2.2.1 and 2.2.2. To describe this condition formally, let 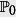 denote any probability measure under the global null hypothesis of no informative SNPs in {1, …, *d*} at all. We assume that the following condition holds true: Let p^*^ denote the smallest of the *k p*-values corresponding to the positions picked by the SVM method for which the null hypothesis of no association between SNP and trait is true. Regarding p^*^ as a random variable, assume that its distribution under the true data-generating distribution 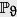 (which is unknown) is stochastically not smaller than under 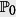.

The distribution under 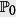 of the *k p*-values corresponding to positions chosen by applying the SVM method is now estimated by the resampling procedure given below as Algorithm 2. The algorithm repeatedly assigns a random permutation of the phenotypes *y*_*π*(1)_, …, *y*_*π*(*n*)_ to the observed genotypes *x*_1*_, …, *x*_n*_. The empirical lower *α*-quantile of the smallest of these *k p*-values is then a valid choice for t^*^ in the sense that the *FWER* for the entire procedure defined by Algorithm 1 is bounded by *α*.


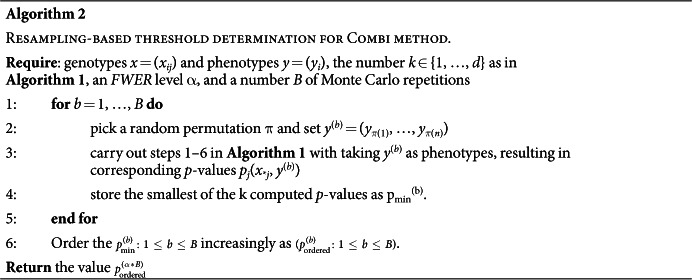


Note that the choice *k* = *d* leads to skipping the SVM step and arriving at the popular *MinP procedure*, originally proposed in Westfall and Young^23^. Following the argumentation in Dudoit and van der Laan^28^, it is also possible to control the generalized *FWER* (*gFWER*) with parameter *l* ≥ 1with the aforementioned resampling scheme as well as the *ENFR*. For *gFWER* control with parameter *l*, one has to consider the (*l* + *1*)th-smallest of the re-sampled *p*-values, instead of 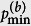 in Algorithm 2. For *ENFR* control, one has to store all B * k computed *p*-values and determine the *p*-value threshold that leads to an average number of rejections (over the *B* Monte Carlo repetitions) which matches the desired *ENFR* level. Moreover, so-called augmentation techniques^28^ can be utilized to control the *false discovery rate* (*FDR*) instead of the *FWER*.

## Results

### Validation

#### Validation using simulated phenotypes

To assess the performance of the proposed COMBI method in comparison to other methods in a controlled environment, we conducted a number of simulation experiments with *semi-real* data. A block of 10,000 genotypes were taken from real WTCCC data^1^ without breaking linkage, but the phenotypes were synthetically generated according to a known model. This ensures that the “basic truth” is known (allowing us to compute the number of true and false positives for each method in the comparison). We show that COMBI outperforms the most commonly used methods for GWAS on these data sets. For instance, it achieves higher true positive rates for all family-wise error levels than any other method that we have investigated^26^,^29^,^30^, including RPVT. In comparison to RPVT, the gain in true positive rate is up to 80%. For a detailed description and analysis of the semi-real data simulations, see Supplementary Section 2.

#### Validation using WTCCC data

**Figure 2:**
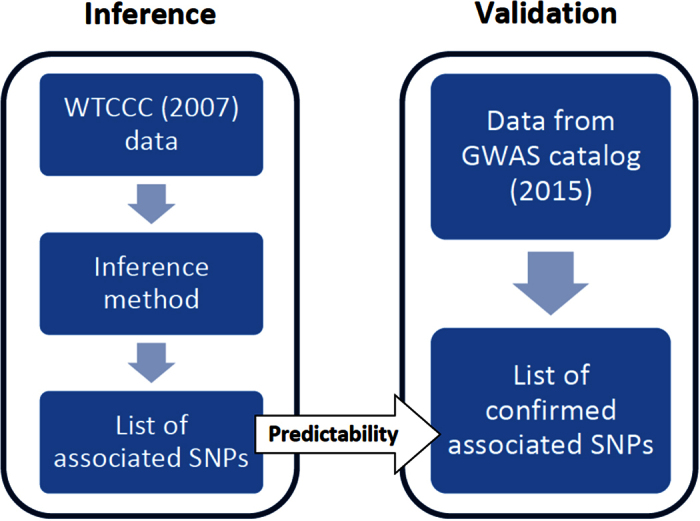
Illustration of validation methodology. After producing a list of associated SNPs via an appropriate inference method (i.e. COMBI or RPVT), the GWAS catalog is used in an independent validation step to confirm or refute those candidate SNPs accessing the predictability of the used inference method.

We then compared the performance of the COMBI method to that of other methods when applied to data from the 2007 WTCCC phase 1, consisting of 14,000 cases of seven common diseases and 3,000 shared controls (see Supplementary Section 3 for further information). In contrast to the simulations described above, the true underlying architecture of the traits under study is largely unknown. Hence, we used replicability in independent studies, one of the standards in the field, as a measure of performance. In summary, we proceeded as follows: the application of some method (for instance, COMBI or RPVT) to the 2007 WTCCC data results in a list of SNPs that are potentially associated with the trait (this is illustrated on the left-hand side of Fig. 2).

We then evaluated this list of potentially associated SNPs for replicability on independent data to obtain the “List of confirmed associated SNPs” (illustrated on the right-hand side of Fig. 2). All studies for the WTCCC diseases included in the GWAS catalog by June 26, 2015 constituted the set of studies examined for replicability. Most of these studies were performed either with larger sample sizes or using meta-analysis techniques and were published after the original WTCCC paper. In a sense, we thus examined how well any particular method, when applied to the WTCCC dataset, is able to make discoveries in that dataset that were actually confirmed by later research using RPVT in independent publications.

Our validation procedure considers a physical window of 200kb around a certain SNP and selects all SNPs with strong LD (R^2^ > 0.8) with the original SNP within that window. It queries the GWAS catalog for those SNPs to find out whether the selected SNPs have any entries. A hit indicates that a GWAS other than the original WTCCC study has since reported this SNP to be associated with the disease. Note that the GWAS catalog only contains SNPs with *p*-values < 10^−5^, meaning that we will miss some hits that are statistically weak but that might be biologically relevant, in the sense that they contribute to the classification of individuals according to phenotypes. For a detailed description of the automatic validation procedure, see Supplementary Section 3.2. With this procedure, methods can be compared by counting the respective number of replicated and non-replicated reported associations.

Regarding significance levels, we aimed to stay as close in line with the original WTCCC study as possible, reporting not only the strong associations at the significance level of 5 × 10^−7^ but also weak associations at 1 × 10^−5^. Within our validation pipeline we considered the full NHGRI GWAS Catalog^7^ with the inclusion criterion of having achieved a *p*-value of 1×10^−5^ in a GWAS. The “*somewhat liberal statistical threshold of p < 1* × *10*^*−5*^ *was chosen to allow examination of borderline associations and to accommodate scans of various sizes while maintaining a consistent approach*”^7^.

We also ensured that the same statistical criterion (control of the *FWER* or the *ENFR*, respectively) was used for all methods, in order to have a fair comparison. This procedure is explained in detail in the Methods Section and Supplementary Section 1.1.1.

#### Stability analysis

In addition, we established an “internal” validation by analyzing the *stability* of the reported associations (cf. Supplementary Section 3.4 for details); this stability measure indicates how well results can be reproduced on another independent sample.

#### Parameter selection

The analysis of WTCCC data required the selection of all free parameters of the COMBI method (e.g. the SVM optimization parameter *C*, the window size *l* of the moving average filter or the filter norm parameter *p*). To this end, the semi-real datasets investigated in Supplementary Section 2 have been used to determine performance changes induced by varying those free parameters. Since our findings were in agreement with related literature and mostly biologically sensible, the optimal settings were assumed to be good choices for the application of the COMBI method to real data. For example, it was found that aggregating SNPs within the filtering step (See Summary of the COMBI method and Fig. 1, the filtering step) based on a filter size of 35 is optimal, which is on the same magnitude as in Alexander and Lange^31^ who find that grouping of SNPs into bins of size 40 helps the performance of their algorithm. The moving average filter of the COMBI method is designed to correct for non-independence of statistical tests within LD blocks. Given the SNP density in the arrays used by the original WTCCC study and LD patterns in the CEU population (1000 Genomes), we estimate that the average LD block (r^2^ > 0.8) will harbor no more than 20–30 SNPs^32^, which supports our findings of setting the filter window size to 35 in the sense that we average-out blocks and conservatively add a bit of noise by potentially smoothing out signals across blocks.

See Supplementary Section 1.1 for a detailed description of the selection of all free parameters of the COMBI method.

Some parameters of the COMBI method could not be investigated within the simulation study, but had to be chosen manually for the WTCCC data. The decision to train the SVM separately on each chromosome was one of those tuning steps, as genome-wide training is very time and memory consuming on the one hand, and can only improve performance marginally on the other hand, as intergenic correlations between chromosomes are very rare.

Another parameter that was chosen manually was the number of active SNPs in one chromosome, i.e. the parameter *k* of The Screening Step presented in the Methods Section, which was set to 100 SNPs per chromosome after careful consideration. This choice is admittedly a wide, arbitrary upper bound for the number of SNPs that can present a detectable association with a given phenotype. Currently, the maximum total number of SNPs (not independent signals) associated with any phenotype is ~450 for human height and 180 for Crohn’s Disease (GWAS Catalog, accessed June 2015), so with *k* = 100 per chromosome one is well within what current evidence would support. After all, for future applications of COMBI *k* is a tuning parameter which has to be chosen by the researcher according to the assumed number of relevant loci.

The choice of exact values for all parameters will probably need to be adapted for each particular phenotype or disease under study, since they will have different genetic architectures and distribution of effect sizes^4^,^9^. For this manuscript and in order to provide a comprehensive and comparable set of results across many diseases we employed a unique set of parameter values supported by the results of our simulation study and other findings in related literature.

### Manhattan plots and descriptive results

**Figure 3:**
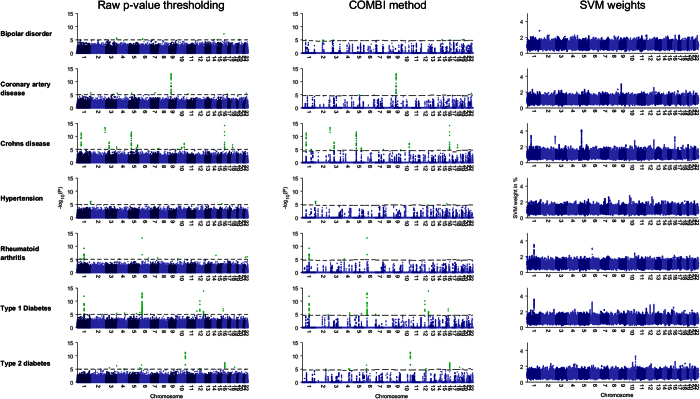
Genome-wide scan for seven diseases. Manhattan plots for all seven diseases resulting from the standard RPVT approach and the COMBI method as well as the SVM weights. We plot −log_10_ of the χ^2^ trend test *p*-values for both COMBI and RPVT and the corresponding SVM weights against position on each chromosome. Chromosomes are shown in alternating colours for clarity, with significant *p*-values highlighted in green. Please note that for the RPVT, the threshold indicated by the horizontal dashed line is fixed *a priori* genome-wide. For the COMBI method, it was determined chromosome-wise via the permutation-based threshold over the whole COMBI procedure. All panels are truncated at −log_10_ (*p*-value) = 15, although some markers exceed this significance threshold.

Figure 3 displays Manhattan plots for all seven diseases resulting from the standard RPVT approach (left) and the COMBI method (center) as well as the SVM weights (right). The center and right graph illustrates that the COMBI method discards SNPs with a low SVM score (cf. “The screening step” in Summary of the COMBI method and Fig. 1). Hence, the *p*-values for such SNPs are set to one without performing a statistical test, thereby drastically reducing the number of candidate associations. In contrast, the RPVT method results in *p*-values based on a formal significance test for every SNP, where many of these *p*-values are small and produce a lot of statistical noise. SNPs that show genome-wide statistical significance are highlighted in green in the left and right panel. For the standard RPVT, the threshold indicated by the horizontal dashed line is fixed *a priori* genome-wide. For the COMBI method, however, it was determined chromosome-wise via the permutation-based threshold over the whole COMBI procedure described in the Methods Section and Supplementary Section 1.1.1 to match the expected number of false rejections of RPVT.

In Table 2, we present all significant associations reported by the COMBI method. Associations with a raw *p*-value > 10^−5^ were not reported in studies using only RPVT. If they are selected by the COMBI method, we consider them to be new findings and highlight them in grey. The last column of Table 2 indicates whether the reported associations were validated (i.e., were reported as significant in at least one independent study published *after* the WTCCC). The COMBI method finds 46 significant locations. 34 of these 46 significant locations have a *p*-value below 10^−5^ and were thus also found by the RPVT approach.

Crucially, our COMBI method found 12 *additional* SNPs. Out of these, ten (>83%) have already been replicated in later GWAS or meta analyses. The COMBI discoveries that have been replicated independently using individual SNP testing are for bipolar disorder rs2989476 (Chr. 1), rs1344484 (Chr. 16), rs4627791 (Chr. 3), and rs1375144 (Chr. 2); for coronary artery disease rs6907487 (Chr. 6) and rs383830 (Chr. 5); for Crohn’s disease rs12037606 (Chr. 1), rs10228407 (Chr. 7), and rs4263839 (Chr. 9) and for type 2 diabetes rs6718526 (Chr. 2). Given the current debate on the replicability of GWAS findings obtained by single-SNP analyses^33^, it is remarkable that GWAS studies published later had already replicated more than 83% of novel SNPs the COMBI method detected by reanalyzing data published in 2007.

Two out of the 12 SNPs with *p*-values exceeding 10^−5^ had not yet been reported in any GWAS or meta analyses as being associated with the corresponding diseases. Those are rs11110912 (Chr. 12) for hypertension and rs6950410 (Chr. 7) for type 1 diabetes. SNP rs11110912 was included in the original WTCCC analysis, but a *p*-value higher than 10^−5^ was obtained (1.94 × 10^−5^)^1^, so it was not collected in the GWAS Catalog. SNP rs6950410 has been detected as associated to multiple complex diseases^34^. Regarding the biological plausibility of these two SNPs, we examined a number of functional indicators to assess their potential role in disease. In particular, we explored the genomic regions in which they map and their potential roles as regulatory SNPs, status as eQTLs, and role in Mendelian disease. Overall, there is no strong evidence of functional roles (see Supplementary Section 3.5) but SNP rs11110912 (Chr. 12), for which COMBI suggested a link to hypertension, is an intronic SNP mapping on a gene, MYBPC1, that has been previously linked to familial hypertrophic cardiomyopathy, suggesting that COMBI has given rise to another interesting true positive finding.

### GWAS catalog validation results – results obtained by the COMBI method are better replicated than those obtained by RPVT

**Figure 4:**
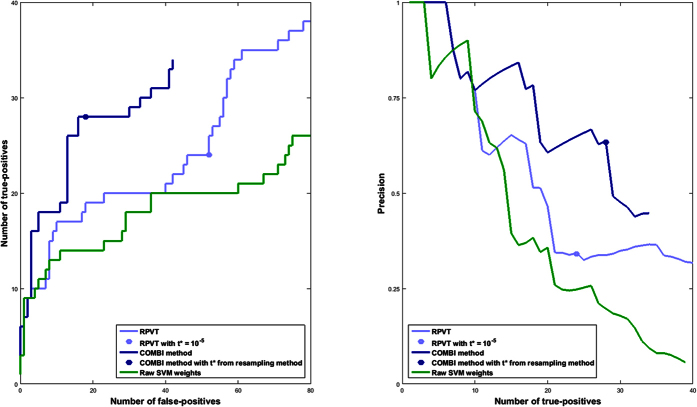
ROC and PR curves of the RPVT approach, the COMBI method and raw SVM weights using the independent validation pipeline as an indicator of replicability. The results of all seven diseases have been pooled. The curves have been generated based on the replication of SNPs according to the GWAS catalog. Replicated reported associations are counted as true positives, and non-replicated associations as false positives. Note that the COMBI lines end at some point and the RPVT and the raw SVM lines continue. At the endpoint of the COMBI curve all SNPs selected in the SVM step are also significant in the statistical testing step; *i.e*. if one wanted to add just one more SNP to the list of reported associations, all other SNPs would also become significant, as they have a *p*-value of 1. The points on the RPVT and COMBI lines represent the final results of the two methods when applying the corresponding significance thresholds and are described in more detail in Table 3.

The COMBI method also outperforms the RPVT approach for different type 1 error levels. Figure 4 shows the receiver operator characteristic (ROC) and precision-recall (PR) curves that have been generated based on the replication of SNPs according to the GWAS catalog (here, due to absence of basic truth knowledge, replicated reported associations are counted as true positives, and non-replicated associations as false positives). As the dark blue lines are consistently above the light blue lines, the COMBI method achieves both higher numbers of *true positives* (*i.e*. higher true positive rate (TPR)) as well as a higher *precision* (proportion of replicated associations amongst the SNPs classified as associated with the trait) for given numbers of *false and true positives* (*i.e*. lower false positive rate (FPR)) than RPVT for almost all levels of error. For comparison, we show also the result achieved when selecting SNP based on the highest SVM weights in absolute value (after filtering). The results show that discarding either one of the two steps in the COMBI method (machine learning or statistical testing step) will lead to a decrease in performance.

We now investigate the points on the curves that correspond to the application of t* = 10^−5^ in the case of RPVT and to the value of t^*^ resulting from the permutation-based method in the case of the COMBI method (described in the Methods Section) in more detail. See Table 3 for the numbers corresponding to those points. A total of 78 SNPs were found to be significant with RPVT, since it only performs the statistical testing step, and 46 with the COMBI method, which has the additional layer of the machine learning screening step prior to the statistical testing.

Although the COMBI method finds fewer SNPs, the number of replicated SNPs is greater (28 in contrast to 24 of RPVT). The COMBI method also classifies only 18 of the unreplicated SNPs as associated with the trait (yielding a precision of 61%). This is in contrast to RPVT, which classifies 52 of the unreplicated SNPs as associated with the trait (yielding a precision of only 32%). In other words, if both methods are calibrated with respect to the same type I error criterion, the COMBI method reports significantly more replicated associations (Fisher’s exact test *p*-value of 0.0014).

### Stability results – COMBI method is more stable than RPVT

From simulations considering internal stability, we found that the COMBI method produces more stable results than RPVT; cf. Supplementary Section 3.4 for details.

### Runtime analysis and implementation details

The COMBI method is implemented in Matlab/Octave, R and Java as a part of the GWASpi toolbox 2.0 (https://bitbucket.org/gwas_combi/gwaspi/). The complete method is available in all these programming languages. The implementation for Matlab/Octave is cluster oriented and uses libLinear^35^. The Java implementation is desktop computer oriented and makes use of the following packages: libLinear^35^, libSVM^36^ and apache commons math^37^. Finally, the R implementation requires LiblineaR^38^, qqman^39^, data.table^40^, gtools^41^ and snpStats^42^.

The runtime of the method depends on a variety of factors such as available cluster memory, hardware resources and operating system. For this analysis we have run the method with the Matlab/Octave implementation on the following technical platform: 40 * Intel(R) Xeon(R) CPU E5-2650 v3 @ 2.30GHz 64bit, 128GB RAM, Ubuntu 14.04.4 LTS (GNU/Linux 3.13.0–79-generic x86_64), GNU Octave version 3.8.1. The analysis of WTCCC’s data on Crohn’s disease chromosome 18 (assuming calculations on more chromosomes can be computed in parallel if necessary) took 9h 15min and 24s. See Supplementary Section 3.7 for a more detailed runtime analysis.

## Discussion

Several related machine learning methods have been successfully used in the context of statistical genomics. These approaches can be classified into two groups:

1. Methods that construct a model from genetic data in order to carry out accurate predictions on a phenotype^43^,^44^,^45^,^46^,^47^,^48^,^49^,^50^,^51^,^52^,^53^,^54^,^55^,^56^.
2. Methods that use machine learning to construct a statistical association test or rank genetic markers according to their predicted association with a phenotype^30^,^31^,^57^,^58^,^59^,^60^,^61^,^62^,^63^,^64^,^65^,^66^,^67^.

The set of papers that fall into the first category study the predictive performance of penalized regression and classification models including support vector machines^16^,^17^,^18^, random forests^68^, and sparsity-inducing methods such as the elastic net^69^ on various complex diseases (including the ones studied here), showing that machine learning methods such as SVMs – if appropriately applied - can perform well at predicting disease risks. See Supplementary Section 3.3, where we compare the prediction performance of various methods on the WTCCC data.

However, the main point of interest of the present contribution does not lie in risk prediction but rather in the identification of regions associated with diseases. The COMBI method should thus be compared to true alternative methods that stem from the second category, some of which include two-stage approaches first performing statistical testing and then machine learning to refine the set of predicted associations^30^,^57^. These approaches, however, are unable to identify correlation structures of SNPs that have been excluded in the first step and neither method is validated on real data in terms of a comparison to the GWAS database. Similarly, Pahikkala *et al*.^59^ and He and Lin^60^ develop methods for ranking genetic markers based on the sure independence screening strategy^70^ and stability selection analyzing only one SNP at a time. Recently the approach has been extended to detect gene-to-gene interactions by Li *et al*.^71^, but neither of the methods have been validated on independent external studies.

Another approach is by Alexander and Lange^31^, who apply the stability selection method of Meinshausen and Bühlmann^58^ to the WTCCC data set to rank SNPs according to their predicted association with a phenotype. The authors find that stability selection effectively controls the *FWER* when applied to GWAS data but suffers a loss of power, while at the same time rendering conservative results.

The work that is probably most closely related to the present research is the two-step algorithm by Wasserman and Roeder^27^ (and the extension by Meinshausen *et al*.^26^), who split the data into two equal parts performing marker selection on the first part and then testing the selected markers on the second part. See Supplementary Section 2.2.4 for a detailed description of this approach.

In order to investigate and compare performance of the COMBI method to other machine learning approaches, the work of Roshan *et al*.^30^, Wasserman and Roeder^27^ and Meinshausen *et al*.^26^ are selected as representative baseline methods. In Supplementary Section 2.2.4, we show that the COMBI approach outperforms all of these methods on semi-real data.

An important and very closely related recent method by Lippert *et al*.^14^,^72^ aims to identify putative significant disease-marker associations using two approaches based on linear mixed models (LMMs): a univariate test and a test for pairwise epistatic interactions. LMMs, like COMBI, address the issue of population stratification in GWAS, cf. Mimno *et al*.^73^. However, in contrast to COMBI, they still test SNPs (or pairs of SNPs) individually one after the other and thus potentially lose detection power. Another possible shortcoming of LMMs and related methods over SVMs is that they are more tailored for regression and not binary classification. For a comparison of COMBI with Lippert *et al*.^14^,^72^ on real WTCCC data see Supplementary Section 3.6. Recently their approach has been extended for disease risk prediction (Rakitsch *et al*.^56^) and related approaches have been proposed by Loh *et al*.^74^ and Song *et al*.^75^ suffering the same drawbacks as discussed above.

An extension of LMMs to multivariate cases was developed by Zhou and Stephens^76^, but has not yet been applied to WTCCC. Fitting LMMs to multiple phenotypes provides no novel insight into analyzing multiple genotypes/SNPs at once, which is the issue COMBI addresses.

Our approach can be extended to explore a number of different research directions by substituting one of the two steps of the algorithm with other suitable procedures. Thus, one could either apply other machine learning prediction methods (as mentioned above) instead of training an SVM in the first step of the COMBI method. For example, the SVM training could be replaced by a SNP selection by random forests or component-wise boosting. Alternatively, one could perform a different statistical test in the final step of the COMBI method, such as procedures correcting for population structures or other confounding factors^72^,^77^. These alternatives are possible options for future research (and some have been implemented in the literature), however, COMBI performs better than any of the other machine learning methods we compared it to (Supplementary Section 2.2.4).

COMBI also seems to perform better than other state-of-the-art methods for univariate analyses. For instance, a recent method by Lippert *et al*.^14^ aims to identify putative significant disease-marker associations from the WTCCC data using two approaches based on linear mixed models: an univariate test and a test for pairwise epistatic interactions. When their univariate method results are checked against the same validation criteria that we used for COMBI, it turns out that our method reports 17 more true positives (4.4 times more positives) for the three diseases for which their univariate method reports at least one hit (Supplementary Section 3.6).

The COMBI method also holds great potential for testing pairwise SNP-trait associations, as it drastically reduces the number of candidate associations by selecting a subset of the most predictive SNPs in the machine learning step. Again, a comparison against the method Lippert *et al*.^14^ propose for detecting epistatic interactions, is favorable to COMBI (see Supplementary Section 3.6). In future work we will extend the COMBI method to a regression setup where the phenotype is not binary.

To summarize, we proposed a novel and powerful method for analyzing GWAS data that is based on applying a carefully designed machine learning step that is tailored to the GWAS data before applying a classical multiple testing step. Certain machine learning models, in particular appropriately designed linear SVMs, take high-dimensional correlation structures into account and thus implicitly incorporate interactions between different loci. A subset of predictive candidate SNPs is extracted within the machine learning step. The *p*-values corresponding to association tests are then thresholded for these candidate SNPs in a subsequent statistical testing step. The COMBI method was shown to outperform the RPVT approach both on controlled, semi-real data and on data from the WTCCC 2007 study, for which reported associations were validated by their replicability in external later studies. The empirical analysis showed a significant increase in detection power for replicated SNPs, while yielding fewer unconfirmed discoveries. Two new (as yet unreplicated) candidate associations were reported.

## Additional Information

**How to cite this article**: Mieth, B. *et al*. Combining Multiple Hypothesis Testing with Machine Learning Increases the Statistical Power of Genome-wide Association Studies. *Sci. Rep*. **6**, 36671; doi: 10.1038/srep36671 (2016).

**Publisher's note:** Springer Nature remains neutral with regard to jurisdictional claims in published maps and institutional affiliations.

## Supplementary Material

Supplementary Information

## Figures and Tables

**Table 1:** Tabular representation of single SNP data.

	A_1_A_1_	A_1_A_2_	A_2_A_2_	∑
*Y* = +1	*n*_11_	*n*_12_	*n*_13_	*n*_1._
*Y* = −1	*n*_21_	*n*_22_	*n*_23_	*n*_2._
∑	*n*_0.1_	*n*_0.2_	*n*_0.3_	*n*

Single SNP data are summarized in categories according to phenotypes (cases, Y = +1, and controls, Y = −1) and genotypes (A_1_A_1_, A_1_A_2_ and A_2_A_2_). The numbers n_ik_ denote the numbers of individuals within the corresponding groups. n is the total number of subjects in the study.

**Table 2:** Association analysis of the SNPs reaching genome-wide significance applying the COMBI method.

Disease	Chromosome	Identifier	χ^2^ *p*-value	SVM weight	*p*-value < 10^−5^ in at least one external GWAS or meta-analysis	References (PMID)
Bipolar disorder (BD)	1	**rs2989476**	1.05e-05	0.0141	YES	19416921
	2	**rs1375144**	1.26e-05	0.0146	YES	21254220
	2	rs7570682	1.77e-06	0.0150	YES	21254220
	3	**rs4627791**	1.18e-05	0.0150	YES	21254220
	14	rs11622475	8.02e-06	0.0235	YES	21254220
	16	**rs1344484**	1.10e-05	0.0245	YES	21254220
	9	rs7860360	1.82e-06	0.0174		
	20	rs3761218	7.15e-06	0.0243	YES	21254220
Coronary artery disease	5	**rs383830**	1.35e-05	0.0174	YES	21804106
(CAD)	6	**rs6907487**	1.22e-05	0.0145	YES	17634449
	9	rs1333049	1.12e-13	0.0262	YES	21606135
	22	rs688034	2.75e-06	0.0287		
Crohn’s disease (CD)	1	rs11805303	6.35e-12	0.0234		
	1	**rs12037606**	1.02e-05	0.0142	YES	17554261
	2	rs10210302	4.52e-14	0.0224	YES	23128233
	3	rs11718165	2.04e-08	0.0163	YES	21102463
	5	rs6596075	3.11e-06	0.0168		
	5	rs17234657	2.42e-12	0.0305	YES	18587394
	7	**rs10228407**	1.08e-05	0.0160		
	9	**rs4263839**	1.61e-05	0.0201	YES	21102463
	10	rs10883371	5.23e-08	0.0227	YES	21102463
	16	rs2076756	7.55e-15	0.0361	YES	21102463
	18	rs2542151	1.93e-07	0.0246	YES	18587394
Hypertension (HT)	1	rs2820037	7.41e-07	0.0155		
	12	**rs11110912**	1.58e-05	0.0197		
	15	rs2398162	6.01e-06	0.0230		
Rheumatoid arthritis	1	rs6679677	<1.0e-15	0.0243	YES	20453842
(RA)	4	rs3816587	7.28e-06	0.0163		
	6	rs9272346	7.38e-14	0.0239		
Type 1 diabetes (T1D)	1	rs6679677	<1.0e-15	0.0247	YES	19430480
	2	rs231726	1.43e-06	0.0129		
	4	rs17388568	3.07e-06	0.0175	YES	21829393
	5	rs17166496	5.97e-06	0.0148		
	6	rs9272346	<1.0e-15	0.0792	YES	18978792
	7	**rs6950410**	1.03e-05	0.0172		
	12	rs17696736	1.55e-14	0.0223	YES	18978792
	12	rs11171739	8.36e-11	0.0244	YES	19430480
	16	rs12924729	7.86e-08	0.0285	YES	17554260
Type 2 diabetes (T2D)	2	**rs6718526**	1.00e-05	0.0159	YES	20418489
	4	rs1481279	9.44e-06	0.0173		
	4	rs7659604	9.61e-06	0.0175		
	6	rs9465871	3.38e-07	0.0162		
	10	rs4506565	5.01e-12	0.0267	YES	23300278
	12	rs1495377	7.21e-06	0.0196		
	16	rs7193144	4.15e-08	0.0293	YES	22693455
	18	rs1025450	1.98e-06	0.0271		

For all seven diseases we present SNPs reaching genome-wide significance along with their rs-identifier, corresponding chromosome, χ^2^ trend test *p*-value, SVM weight and the result of the validation pipeline indicating whether the SNP has been found significant with a *p*-value < 10^−5^ in at least one external GWAS or meta-analysis. PMID references of those studies are given in the last column. SNPs that do not show genome-wide significance in the case of RPVT are highlighted in bold case.

**Table 3:** Empirical evaluation of the performance of the COMBI method on the WTCCC data, relative to that of basic RPVT.

	Number of SNPs reaching significance applying	
	RPVT	COMBI Method
SNPs that have achieved <10^−5^ in at least one external study	24 (32% precision)	28 (61% precision)
SNPs that have not achieved <10^−5^ in an external study	52 (68% error)	18 (39% error)
Overall	76	46
*p*-value (one-sided Fisher’s exact test)	0.0014	

The table represents the information given by the points on the RPVT and COMBI lines in Fig. 4. The final results of the two methods when applying the corresponding significance thresholds are shown. At significance threshold t* = 10^−5^, COMBI achieves 28 SNPs recall at precision 61%, while RPVT achieves a recall of only 24 SNPs at precision 32%.
