# Supplementary material for: Combining Multiple Hypothesis Testing with Machine Learning Increases the Statistical Power of Genome-wide Association Studies
Source: Sci Rep. 2016 Nov 28;6:36671. doi: 10.1038/srep36671 (PMC5125008; doi:10.1038/srep36671)
Supplement: Supplementary Information [file srep36671-s1.pdf]

Supplementary Material

Combining Multiple Hypothesis Testing with Machine Learning Increases the Statistical Power of Genome-wide Association Studies

Bettina Mieth, Marius Kloft, Juan Antonio Rodriguez, Sören Sonnenburg, Robin Vobruba, Carlos Morcillo-Suarez, Xavier Farre, Urko M. Marigorta, Ernst Fehr, Thorsten Dickhaus, Gilles Blanchard, Daniel Schunk, Arcadi Navarro & Klaus-Robert Müller

Content of supplementary material

1. Supplementary Methods
  1.1. Parameter Selection
    1.1.1. Determination of type 1 error level α
    1.1.2. Other free parameters
  1.2. Comparing SVM weights with RPVT *p*-values
2. Simulation study on controlled phenotypes
  2.1. Simulation data
  2.2. Results
    2.2.1. COMBI method vs. standard RPVT
    2.2.2. Conservativeness of the *FWER* control
    2.2.3. Further experiments
    2.2.4. Comparison to other state of the art methods
3. Analysis of real data (WTCCC, 2007)
  3.1. Data preparation
  3.2. Automatic validation procedure
  3.3. Prediction performance
  3.4. Stability analysis
  3.5. Functional study of two non-replicated SNPs
  3.6. Comparison to Fast-LMM models
  3.7. Runtime analysis and implementation details
References of supplementary material

1. Supplementary Methods

1.1. Parameter Selection

1.1.1. Determination of type 1 error level α

Here, we discuss how to determine the type 1 error level α to be used in **Algorithms 1** and **2** presented in the **Methods Section** of the main text when applying the COMBI method to the WTCCC data[1]. When comparing the performance of the COMBI method with that of RPVT, α should correspond to the error level applied in the original WTCCC study[1]. In that case, $t^* = 5*10^{-7}$ was determined to be a reasonable threshold for type 1 error control in RPVT stating that, “the posterior odds in favor of a ‘hit’ being a true association would be 10:1” using this threshold[1]. A sound upper bound on the expected number of false rejections (*ENFR)* level that this threshold implies is obtained by calculating the empirical distribution of p-values using the Westfall-Young[2] procedure. It turned out that the threshold of $t^* = 5*10^{-7}$ will, on average, produce 0.17 non-replicated discoveries per disease, or 1.19 for all seven. Out of the 24 SNPs reported in WTCCC at $t^* = 5*10^{-7}$, only one can be expected to be a false positive, which corresponds to a true-to-false-positives ratio of 23:1.

WTCCC also reported SNPs at the level $t^* = 10^{-5}$, stating that “if we relax the significance threshold by a factor of ten […], the posterior odds that a ‘hit’ is a true association would also be reduced by a factor of ten.”[1] The relaxed threshold of 10^{-5} would thus refer to posterior odds of 1:2. According to our simulations, the controlled number of non-replicated discoveries to be expected is 3.32 per disease on average. This suggests that out of the 82 loci reported by the WTCCC at $t^* = 10^{-5}$, we can expect that approximately 23 are false positives, corresponding to an actual rate of true-to-false-positives of ~ 3:1.

To compare the performance of the COMBI method with that of RPVT in a fair way, we consequently calibrated the COMBI method (using the Westfall and Young-type procedure described in **Algorithm 2** presented in the **Methods Section** of the main text) in a way such that the number of non-replicated discoveries is bounded by 3.32 per disease (using the

augmentation for *ENFR* control of both algorithms). It should be noted that, when investigating the performance of the COMBI method with semi-real data simulations, we observed that the COMBI method produces only approximately 20% of the number of type 1 errors one is aiming to control for (See **Supplementary Section 2.2.2.**). Although it is not known whether this relation is true in the case of real data, we could expect still substantially fewer errors than what the calibration aims for, *i.e.*, around 0.664 instead of 3.32 per disease, if the data-generating distribution is as in the simulations.

1.1.2. Other free parameters

In this section, we discuss how we chose the values of the free parameters of the COMBI method for the investigation of the WTCCC data presented in the **Methods Section** of the main text. To this end, the semi-real datasets investigated in **Supplementary Section 2.** where used to determine performance changes induced by varying the free parameters of the COMBI method. The optimal settings were assumed to be good choices for the application of the COMBI method to real data.

As described in **Problem Setting and Notation** in the **Methods Section** of the main text, the genotypic feature encoding method was applied, where all features where normalized such that the 6th centered moments were all one (this is similar to the common practice of scaling each feature to unit standard deviation).

As we can see from **Equation 1** in **The machine learning step** in the **Methods Section** of the main text, there are a number of parameters to be determined in the training of an SVM**.** During the simulation experiments on the semi-real data sets, the value of the parameter *C* was computed by internal cross-validation, in order to maximize the (estimated) generalization ability of the function *f*. We found no significant effect of this parameter on the performance of the COMBI method. We thus applied a fixed C = 0.00001 for the investigation of the real data, economizing computation time and memory space. A linear L2 regularized L1-loss dual

classifier was applied[3] to solve the SVM minimization problem. We trained the SVM on each chromosome separately, as genome-wide training is very time and memory consuming on the one hand, and can only improve performance marginally on the other hand, as intergenic correlations between chromosomes are very rare.

Reasonable choices of the free parameters of **Equation 3** in the filtering and screening step in the **Methods Section** of the main text were shown to be optimal in the simulation experiments. The window size l of the moving average filter was set to 35, which is in agreement with the results of Alexander and Lange[4], who investigated similar associations. The norm parameter p of the filter was set to 2.

A reasonable upper bound for the number of active SNPs in one chromosome was found to be 100, which is why the parameter k of **The Screening Step** presented in the **Methods Section** of the main text was set to this value.

The simulation experiments also showed better performance when the χ^2 test for trend was applied instead of the Cochran-Armitage test in **The Statistical Testing Step** presented in the **Methods Section** of the main text**.**

1.2. Comparing SVM weights with RPVT *p*-values

A legitimate concern of readers could be that, even if Steps 1 and 2 of COMBI are run independently of each other – in the sense that the w value of a SNP is obtained irrespectively of what the p-value of the same SNP would be in a trend test performed under the usual univariate RPVT setup – high correlations between w and p-values might still occur. If this was the case, the statistical testing step in the COMBI procedure might have to use a more stringent Bonferroni correction. **Supplementary Fig. S1** and **S2** below show that w and the corresponding p-values are not highly correlated. While it is clear that COMBI hits tend to be located in the area with high w values, note that several highly significant SNPs reported in the

WTCCC have moderate *w* values and, vice versa, that not all SNPs with high *w* turn out to be significant. This was expected, since COMBI produces a simultaneous analysis of all SNPs, rather than considering each SNP in isolation.

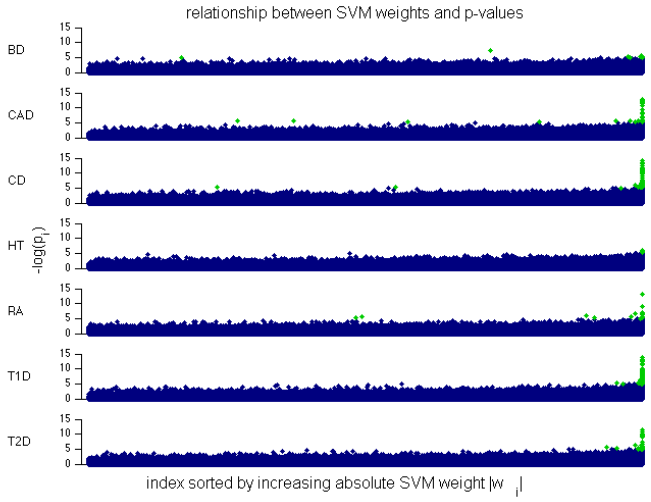

Supplementary Figure S1

Relation between SVM weights and p-values for all 444K SNPs for all seven diseases studied. Following a general trend, higher weights tend to have more significant p-values. However, clear cases with medium or lower weights also turn to be significant, pointing towards the need of a full combined approach (SVM + p-value thresholding step)

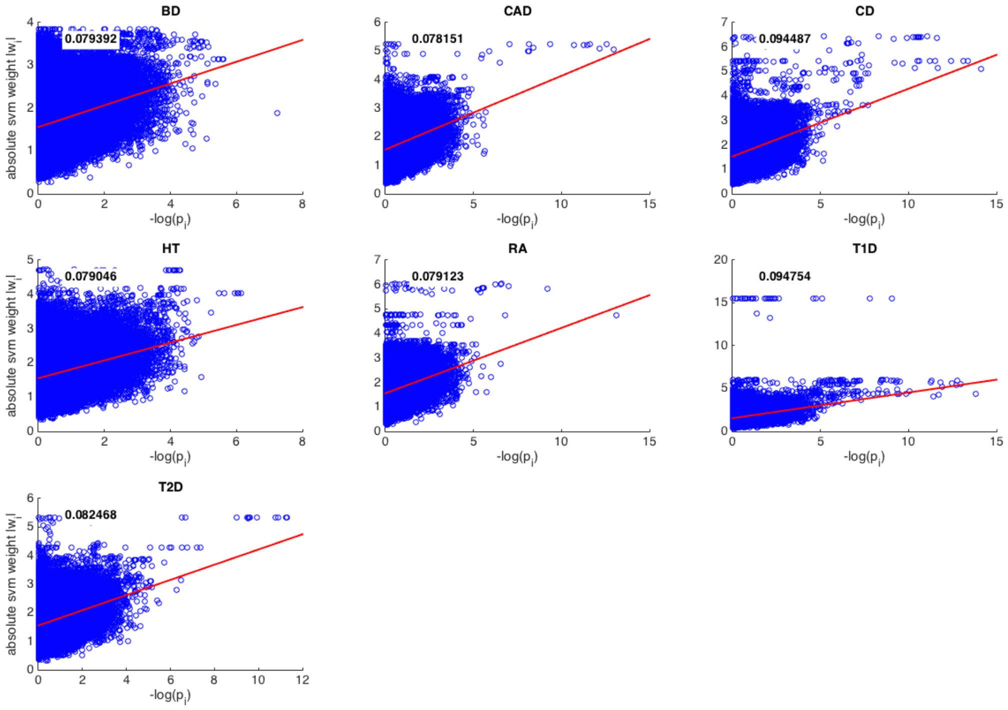

Supplementary Figure S2

Scatter plot of p-values (on –log scale) vs. SVM weights, least-squares regression line, and R^2. As in the former figure, there is a general trend of higher weights having more significant p-values, but there are also many exceptions.

To analyze whether SNPs with high *w* values could be selected as potential hits (thus removing the need of the 2nd step in the COMBI method), we took the top 2,000 SNPs with highest weights and selected a single representative SNP for each of the loci included in the top weights list. We obtained sets of SNPs of the following sizes for each disease: BD (n=115), CAD (117), CD (n=96), Hypertension (103), Rheumatoid Arthritis (106), T1D (85) and T2D (114). We excluded hits already reported by the full COMBI approach (those in Table 2 in the Main Text). All of these sets were evaluated using the validation pipeline and precision-recall and ROC curves can be seen in **Figure 4** of the main text.

2. Simulation study on controlled phenotypes

This section aims to assess the effectiveness of the proposed algorithm and investigating its optimal parameter values *in a controlled environment*, *i.e.*, where we have access to the true phenotypes by synthetically generating them. The genotypes are taken from real WTCCC data to obtain realistic semi-real data sets.

2.1. Simulation data

To this end, we randomly generate 10,000 data sets (*i.e.*, real genotypes and synthetic phenotypes) by repeatedly drawing a random block of 20 subsequent SNPs from chromosome 1 and a random block of 10,000 subsequent SNPs from chromosome 2 of WTCCC's IBD data. The former, smaller block represents the set of informative SNPs to be associated with the phenotype in this experiment, while the latter, larger block constitutes the set of uninformative SNPs. These *noisy* SNPs are placed surrounding the 20 informative loci, which thus are to be found at the positions 5001 to 5020. The phenotypes are synthetically computed, based on only one of the 20 associated SNPs (at position 5010), using the following logistic regression model: to any allele sequence x_{i*} in nominal feature encoding (i.e. $\mathrm{x_{ij}} = \mathrm{m_{ij}}$ where $\mathrm{m_{ij}}$ is the number of minor alleles in SNP j of subject i), the phenotype is randomly assigned according to

$$\mathbb{P}(Y_i = +1 | X_{i*} = x_{i*}) = \big(1 + \exp(-\gamma(x_{i5010} - \mathrm{median}(x_{*5010}))\big)^{-1},$$

where γ is the effect size or noise parameter. Due to the real correlation structure within the set of 20 informative SNPs, generating the phenotypes based on one informative SNP will produce associations of different strengths to all 20 SNPs. Thus, we produce tower structured p-values with realistic covariances; exemplary data sets are shown in **Supplementary Figure S3**.

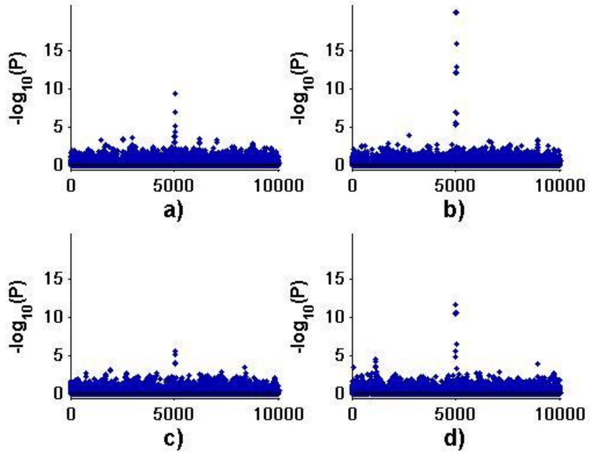

Supplementary Figure S3

p-values of several exemplary semi-real data sets. Note the tower structure induced by the 20 informative SNPs located at the positions 5001 to 5020. While the data sets (a), (b), and (d) present quite strong associations, data set (c) only shows rather minor ones - this is a result of the randomness involved in the generation process.

Note that the associations between the informative SNPs and the labels vary a lot in strength due to the randomness in the generation process, which increases similarity to real genome data sets.

The process of drawing random genotypes and generating the corresponding phenotypes is repeated 10,000 times to generate 10,000 data sets.

An additive heritability model was assumed appropriate for this simulation study for a number of different reasons. Most importantly it is the standard model in the field of SNP effect estimation, genome risk score computation and other related problems[5,6]. This is especially true for the seven diseases that are studied in this work. In the original WTCCC study, they used an additive test as the null model, spotting only a few cases were departure from this additivity was observed[1]. Additive, infinitesimal models have been shown to work well in the research area of quantitative genetics and, indeed, most GWAS hits seem to behave additively[7,8,9]. It is

also the most agnostic model, with less parameters and no need to make any assumptions on values of dominance or complex interactions between loci[5,6]. The investigation of other models for the genetic architecture of a disease could be the subject of future research projects.

2.2. Results

In the following, we report results averaged over the 10,000 data sets realized by the Monte Carlo simulation described above. The proposed COMBI method is compared to the standard RPVT procedure using a Westfall-Young correction as well as the approach of Meinshausen et al.[10] (which itself is an extension of the method of Wasserman and Roeder[11]). As evaluation criteria, we report the family-wise error rate, *FWER*, the expected number of false rejections, *ENFR*, and the true-positive rate, *TPR*. The results are presented in the following section.

2.2.1. COMBI method vs. standard RPVT

The identification of SNPs that have an influence on the probability of having a positive label in the semi-real data sets can be improved by applying the COMBI method, which achieves significantly better results than the ordinary RPVT approach. The superiority of the new method is displayed in **Supplementary Figure S4**, where we compare the power, as measured by the *TPR* (averaged over the 10,000 runs) of the COMBI method to that achieved using the standard RPVT procedure. Those rates can be determined for various levels of *FWER* (shown on the left) and *ENFR* (shown on the right), respectively. It is obvious that the COMBI method achieves greater power for all levels of *FWER* and *ENFR*, which is based on the fact that the selection step of the COMBI method correctly filters out most of the noise SNPs and identifies the informative SNPs accurately.

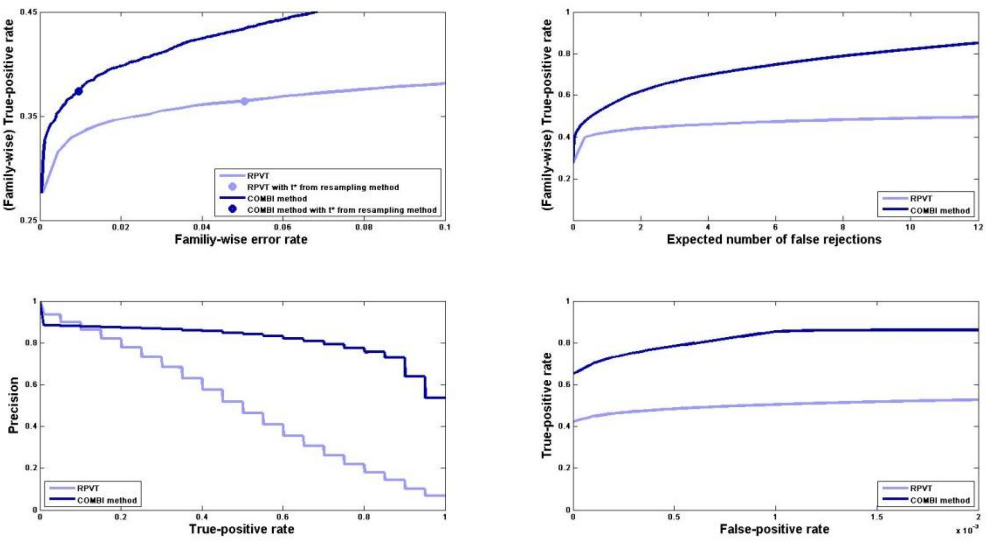

Supplementary Figure S4

Main results of both COMBI and RPVT method applied to the simulated data sets with controlled phenotypes: (a) TPR averaged over 10,000 synthetic data sets as a function of the $FWER$. (b) TPR averaged over 10,000 synthetic data sets as a function of $ENFR$. (c) Precision-Recall curves. (d) ROC curve. The dark blue lines (representing the COMBI method) are uniformly above the light blue ones (representing RPVT) in all plots. The COMBI method thus achieves higher TPR (*i.e.*, multiple power) for all levels of $FWER$ and $ENFR$. The dots in (a) mark measurements of the permutation-based calibration where the corresponding thresholds were calibrated to guarantee a $FWER$ of $\boldsymbol{\alpha \leq 0.05}$. Although the COMBI method in combination with the permutation-based calibration is overly conservative and does not exploit the error rate of 5%, it has greater power than RPVT with permutation-based threshold calibration.

We illustrate an exemplary replication in **Supplementary Figure S5** to provide some intuitive understanding of the advantages of the COMBI method. Not only does the selection step of the COMBI method precisely identify the correct tower, but it also flattens out any noisy SNPs even when - by chance - they achieved considerably high significance. The method thus not only increases the probability of finding the correct tower but also, and potentially more importantly, decreases the probability of falsely selecting a noise tower.

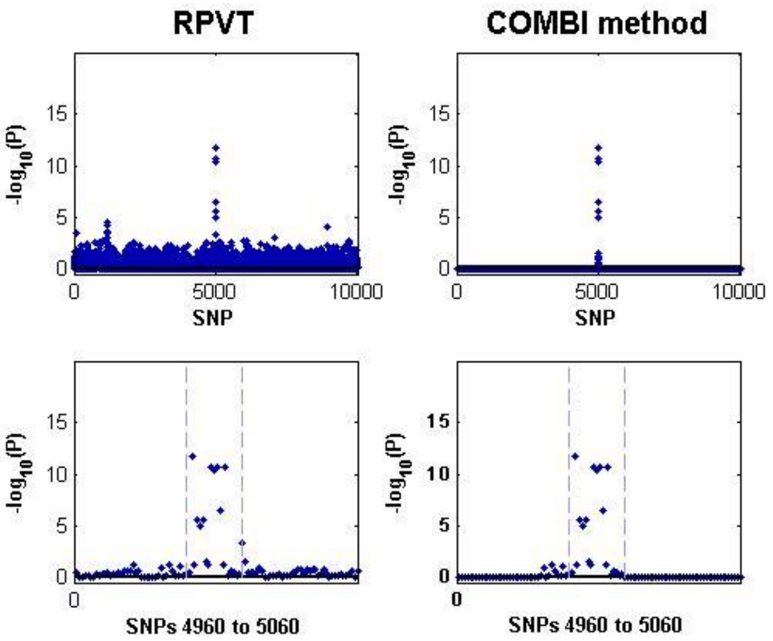

Supplementary Figure S5

An exemplary replication. Plotted are the p-values of the standard RPVT approach on the left and the new COMBI method on the right for all SNPs (top row) and for the 100 SNPs located at 4960 to 5060 (bottom row). On the one hand, there is a very significant tower representing the informative SNPs at positions 5001-5020 (p-values of up to $\mathbf{10^{-13}}$), but on the other there is a considerably large noise tower around the position 1000 with p-values of up to $\mathbf{10^{-5}}$. This tower causes problems in the standard testing approach where it is incorrectly classified as an informative locus. The selection step of the COMBI method, however, precisely identifies the correct tower and flattens out all noise SNPs.

Going back to the averaged results, **Supplementary Figure S4** also shows that the permutation-based threshold calibration based on **Algorithm 2 (**See the **Methods Section** in the main text) yields a rather conservative error rate. The COMBI method does not exploit the full significance level, but makes fewer errors than anticipated. Instead of the previously set error rate of $\alpha \leq 0.05$, a *FWER* of only around 1% is achieved. Even though it is desirable to increase power by simultaneously making more mistakes, i.e. as many as anticipated, it is important to note that the COMBI method has lower error rate and higher power than the RPVT method in combination with the same permutation-based calibration principle. Reasons for the conservativeness of the COMBI method will be investigated in **Supplementary Section 2.2.2**.

To understand the results in more detail, we now investigate the individual data sets. **Supplementary Figure S6** shows for all 10,000 synthetic data sets the level of difficulty of the problem, represented by the power that can be achieved via RPVT, and how well it can be solved using the COMBI method, represented by the gain in *TPR* of the COMBI method over RPVT. In the majority of cases, the COMBI method helps performance, *i.e.*, increases the *TPR*. However, it decreases performance in very few cases (about 3% of the 10,000 data sets). As expected, those cases represent difficult problems with high noise where the baseline *TPR* of RPVT is very low.

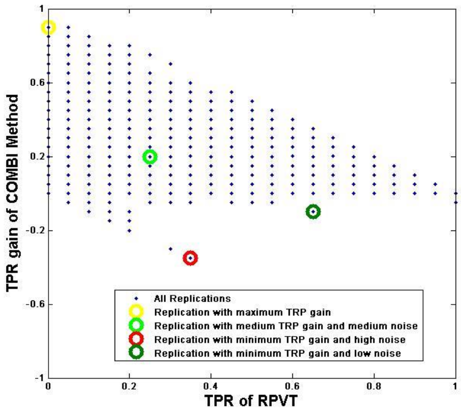

Supplementary Figure S6

The *TPR* gain of the COMBI method is plotted as a function of the *TPR* of RPVT, which is interpreted as a measure of the difficulty of the problem (i.e. level of noise). Each dot represents one replication and indicates how much can be gained in terms of *TPR* by applying the COMBI method instead of RPVT for this specific data set. In most cases, the *TPR* gain is positive, indicating an increase in performance when using the COMBI method. The few cases where power is lost with the COMBI method are characterized by a high level of difficulty in the first place, *i.e*. RPVT *TPR* is low. Four replications are highlighted. Three of them represent special cases with extraordinary characteristics (i.e. maximum *TPR* gain, minimum *TPR* gain with low and high noise) and the fourth represents an average run with medium *TPR* gain and medium noise. See **Supplementary Figure S7** for the individual results of those replications.

In order to investigate the different situations to be encountered in a real world setting, we now analyze a number of special replications. Detailed plots of exemplary runs that either represent an average run (*i.e*. medium *TPR* gain and medium noise) or have extraordinary characteristics (*i.e*. maximum *TPR* gain, minimum *TPR* gain with low and high noise) are shown in **Supplementary Figure S7**. The first row represents the replication with maximum TPR gain where the COMBI method worked extremely well. Although there is a lot of noise and the tower of SNPs associated with the phenotype located at positions 5001-5020 is not very high in this example, the COMBI method finds it accurately. An average replication with medium noise and medium *TPR* gain, *i.e*., where both methods find the tower and the COMBI method can only moderately help performance, is presented in the second row. The third example illustrates that RPVT is sufficient for very easy problems (*i.e*. low noise and high tower yielding minimum *TPR* gain), and that using the COMBI method does not decrease performance. The most crucial case is presented in the last row. In this worst case scenario of a minimum *TPR* gain and high noise, there is an extremely small tower that is very hard to identify. In addition, there is another high-noise tower with very high SVM weights. In contrast to the RPVT approach, which identifies the correct tower, the COMBI method selects the wrong tower. This example shows that the COMBI method selects the wrong towers in very few cases.

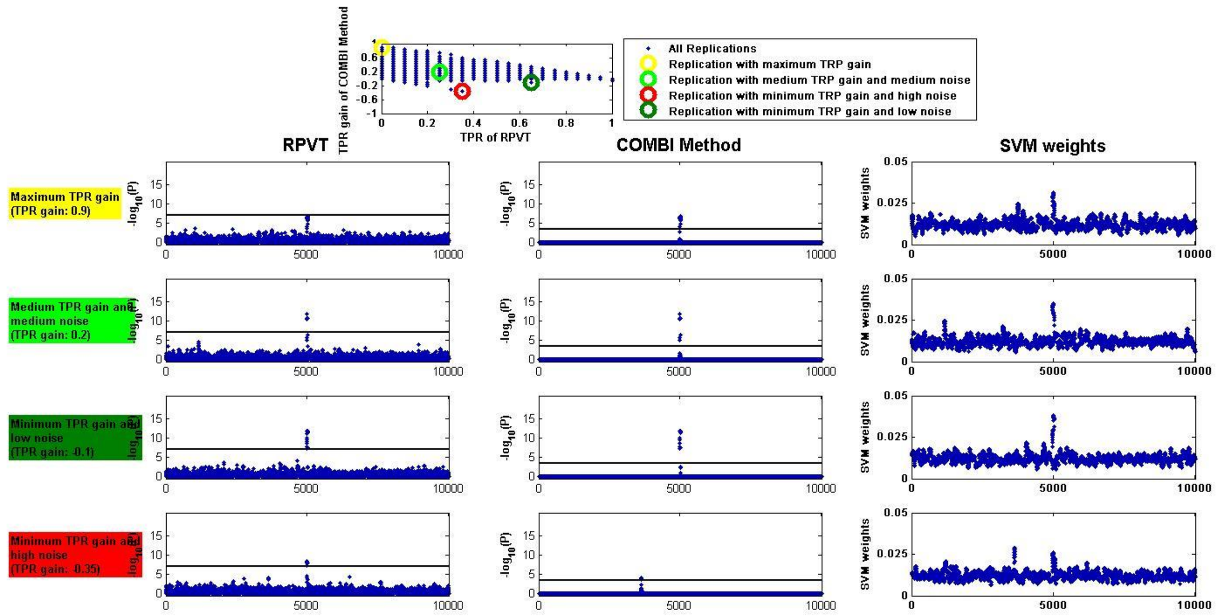

Supplementary Figure S7

Detailed plots of exemplary runs with (1) maximum *TPR* gain, (2) medium *TPR* gain and medium noise, (3) minimum *TPR* gain and low noise and (4) minimum *TPR* gain and high noise. The p-values of the COMBI method and the SVM weights are shown for each replication the p-values of RPVT. The first three replications represent problems where the COMBI method does better or at least as well as the RPVT approach. The last example illustrates that the COMBI method selects the wrong towers in some cases.

We now investigate whether these pathological cases can be identified a priori. As observed in **Supplementary Figure S7**, these cases are characterized by a high noise level, indicating that a very hard problem must be solved. Finding the data sets where an SVM trained for classification of the subjects does not have high accuracy is an intuitive idea. Those cases would be expected to be those where the COMBI method also fails, which is exactly what can be seen when investigating the SVM accuracies for each replication in **Supplementary Figure S8**. The problematic cases -- where power is lost with the COMBI method -- are indeed characterized by a low SVM classification accuracy. These cases can thus roughly be estimated in advance, and a measure of trust in the results of the COMBI method can be given for each replication.

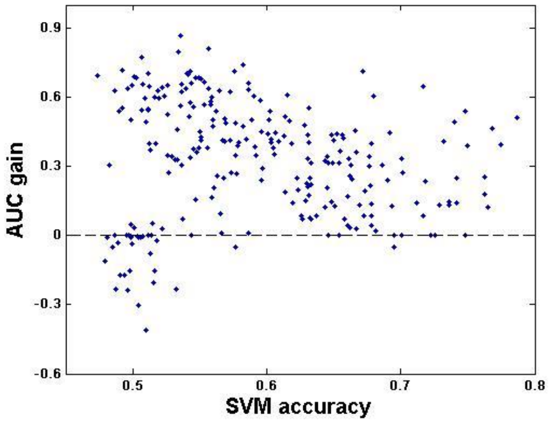

Supplementary Figure S8

For each replication, the *AUC* gain of the COMBI method is plotted against the corresponding SVM accuracy. Negative AUC gain marks the problematic cases where power is lost. It can be seen that the COMBI method only yields a loss in performance in some of the experiments with low SVM accuracy, which means that problematic cases can be identified and a measure of trust can be reported along with the results of the COMBI method for each data set.

2.2.2. Conservativeness of the *FWER* control

Supplementary Figure S4 showed that the COMBI method achieves an overall *FWER* of around 1%, even though the resampling threshold was a priori set to guarantee that *FWER*≤ 5%. We provide an explanation for the conservativeness of the COMBI method in the following section.

An effect that makes the COMBI method conservative is the different number of uninformative (or "*noisy*") SNPs the threshold calibration is based on and eventually applied to. To illustrate this, assume that *k* equals 30, indicating that the 30 SNPs with the highest SVM weights are selected for each replication in the permutation test and p-values are computed for only those. The significance threshold based on these p-values is then determined. As the threshold is calibrated on random labels, it is based on the p-values of 30 uninformative SNPs. However, when we train on real labels when applying the threshold, it is very likely that 20 informative SNPs are selected as part of the 30 highest ranked SNPs. There are thus only 10 spots left for the *noisy* SNPs, which are rejected only if they exceed the threshold. Having 10 instead of 30

noisy SNPs makes an erroneous rejection much more unlikely. Thus, the COMBI method makes fewer mistakes than anticipated and is rather conservative. To validate this hypothesis, we perform a number of experiments where parameter values are altered to achieve the correct *FWER* of 5%. In the first experiment, k is increased in a way that the number of noisy SNPs remains constant. Instead of only selecting k SNPs (noisy or informative), we select the informative SNPs that are amongst the *k* best SVM weights; in addition to that, the best *k* noisy SNPS, i.e., $k_i^* = k + n_{TP,i}$ where $n_{TP,i}$ is the number of true positives among the *k* best SNPs in the i-th replication of the experiment. This should eliminate the effect described above and hence yield a *FWER* closer to that set prior to the permutation test. Applying this slightly modified COMBI method to the 10,000 semi-real data sets leads to a *FWER* of around 5% and thus supports this hypothesis. Note that these modifications can only be applied to data sets where the ground truth, and thus $n_{TP,i}$, is known. To investigate this effect in more detail, we now perform an experiment where we increase the number of selected SNPs, in order to check whether this also yields a less conservative error rate. **Supplementary Figure S9** shows that increasing *k* to a maximum of 1,000 yields a *FWER* of 5%. This is reasonable, as increasing *k* means increasing the fraction of noisy SNPs in the set of SNPs that are picked via the permutation-based calibration procedure. For *k*=1,000, all SNP towers are selected and we simply perform the standard RPVT method which yields a non-conservative *FWER* of 5%. The fewer the selected SNPs, the better the curve and the more conservative the permutation-based calibration. The optimal curve but also most conservative threshold calibration is reached for *k* = 30, which is the parameter chosen for all other applications of the COMBI method. For a lower value of *k*, the *FWER-TPR*-curve is noticeably below the optimal one, and the permutation-based calibration suffers a severe loss in power.

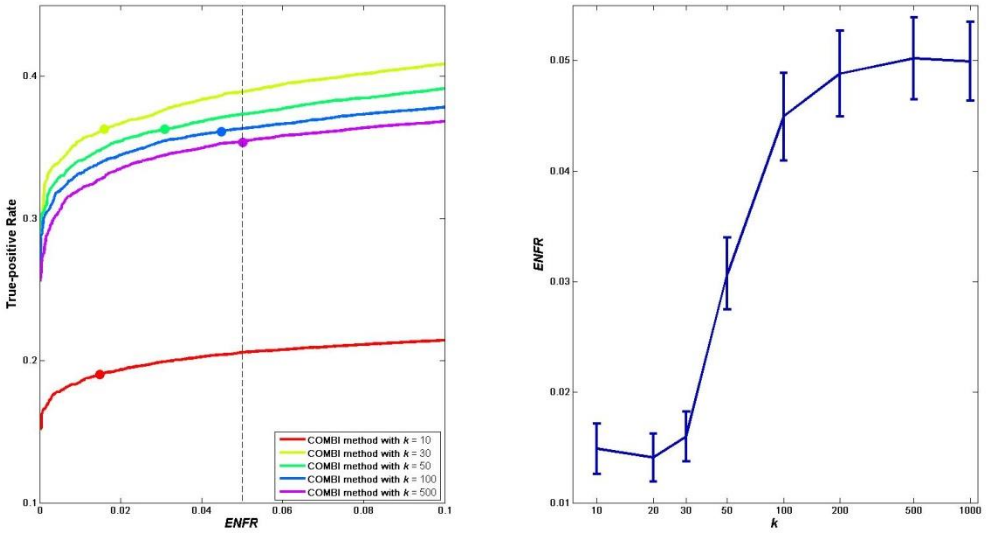

Supplementary Figure S9

Results of the COMBI method for different values of k, the number of SNPs to select in the screening step. (a) TPR is plotted against $ENFR$ for increasing k from 10 to 500. The points represent the results of the COMBI method after applying the permutation-based threshold. The lime green curve of k = 30 is optimal. This is also the parameter value that yields the highest power using the permutation-based calibration (lime green circle). The higher k, the less optimal are the resulting curves (from green over blue to purple), but also the closer is the $ENFR$ of the permutation-based calibration method to the anticipated 5%. Decreasing k below 30 results in a severe loss in power (red curve). (b) Mean and standard deviation of $ENFR$ are shown for different values of k. The maximum error rate which equals the pre-set bound of $\boldsymbol{\alpha \leq 0.05}$ is attained for k=1,000, which corresponds to selecting almost all towers and therefore is almost equivalent to applying RPVT.

The next parameter to be investigated is the noise parameter, which is involved in the data generation process. We expect that for high levels of noise, *i.e.* when there is basically a lack of any kind of real association, the $FWER$ of the COMBI method approaches the expected 5% because the 30 selected SNPs selected are all noisy SNPs. Thus, the threshold is not only based on noisy SNPs but also only applied to noisy SNPs. Observe from **Supplementary Figure S10** that for $\alpha = 0.05,$ the correct $FWER$ is actually achieved for a maximum level of noise. However, the curve is optimal for minimum noise where the identification of true associations is much easier. This experiment supports the hypothesis presented earlier.

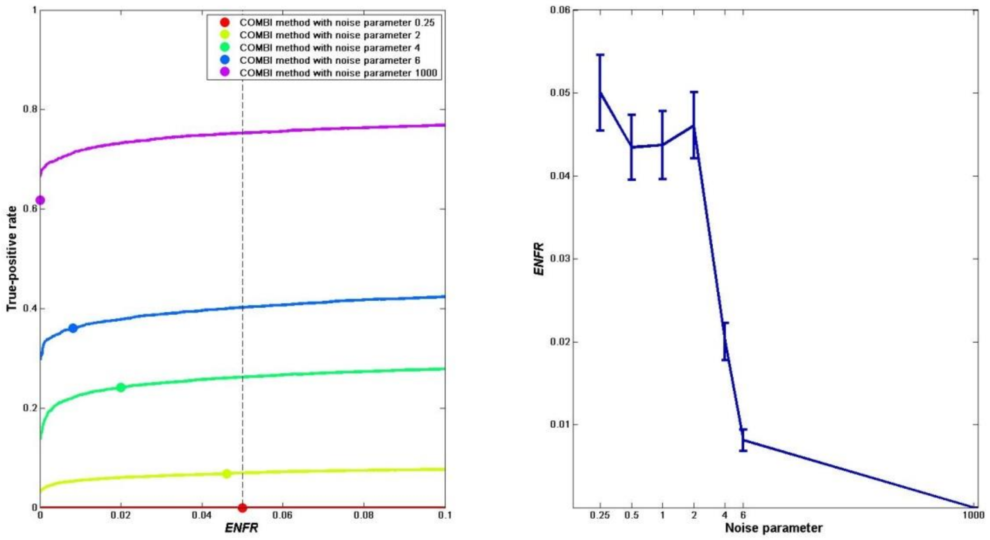

Supplementary Figure S10

Results of the COMBI method for different values of the noise parameters $\boldsymbol{\gamma}$ where the low values correspond to high noise and the high values to low noise in the process of simulating the datasets. (a) *TPR* is plotted against *ENFR* for increasing the noise parameter from 0.25 to 1000. The points represent the results of the COMBI method after applying the permutation test threshold. The curve is optimal for minimum noise (purple curve), which was to be expected as a low level of noise makes true associations easier to detect. (b) Mean and standard deviation of *ENFR* are shown for different values of the noise parameters. The maximum error rate, which equals the pre-set bound of $\boldsymbol{\alpha \leq 0.05}$, is attained for a noise parameter of 0.25, which is almost equivalent to having maximum noise and therefore no informative SNPs associated with the disease.

Observe from **Supplementary Figure S11** that we achieve the error rate for filter length 1 that we would expect after setting it to $\alpha \leq 0.05$ in the permutation test. The error rate decreases and power increases with increasing filter length up to 35. The method yields higher error rates and less power for greater filter lengths. We therefore decided to use a filter length of 35, which yields optimal but conservative results. We learn from these experiments that the proposed method may achieve lower error rates and higher *TPR* than anticipated.

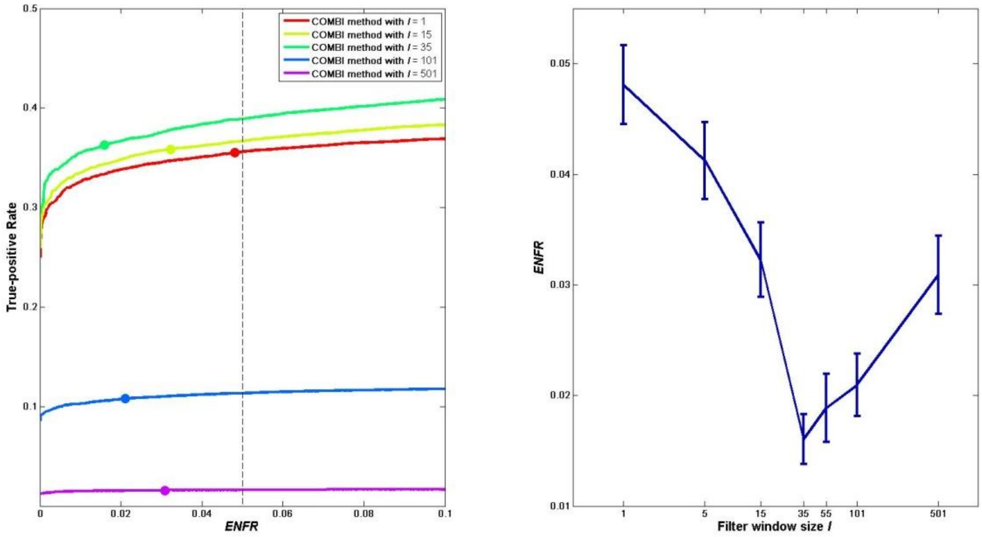

Supplementary Figure S11

Results of the COMBI method for different values of the filter window size *l*. (a) *TPR* is plotted against *ENFR* for increasing *l* from 1 to 501, where the former corresponds to applying no filter at all and the latter to an extremely flattening filter. The points represent the results of the COMBI method after applying the permutation-based threshold. The curve is optimal for a filter window size of 35. This finding is in agreement with what Alexander and Lange[4] found. (b) Mean and standard deviation of *ENFR* are shown for different values of the filter window size *l*. The maximum error rate is attained for filter length 1, which corresponds to applying no filter.

2.2.3. Further experiments

Choosing an optimal SVM parameter using cross-validation-based model selection in each repetition of the Westfall-Young permutation procedure did not result in a higher power of the COMBI method. Performance results for constant and cross-validated C were hardly distinguishable. Thus, time-consuming cross-validation was avoided for C, and a fixed C was used for all further applications of the COMBI method.

2.2.4. Comparison to other state of the art methods

In addition to comparing the COMBI method with the RPVT approach, we investigate here whether slight alterations and simplifications of the COMBI method can achieve the same level of effectiveness and also examine other appropriate state-of-the-art algorithms using semi-real data (See **Supplementary Section 2.** for a description of these semi-real data experiments and the **Discussion Section** of the main paper for a discussion of related machine learning work).

Beginning with the investigation of simplifications of the COMBI method, we now present the results of corresponding simulations. As mentioned earlier, applying a moving average filter to the SVM weights prior to the selection step is crucial. Observe in **Supplementary Figure S12** that the COMBI method cannot increase power or precision of RPVT at all without this filtering step.

To find out whether the filtering step, which improves the performance of the COMBI method, can also be applied to the p-values, p_j, in log-space in order to achieve the same effect, we define $w_j \coloneqq -\log_{10} p_j$ and apply the filter as described in **Summary of the COMBI method** and **Figure 1** (see main text). We employ RPVT in the original p-value space. **Supplementary Figure S12** illustrates that this decreases the performance of the RPVT method and thus cannot reach the effectiveness of the COMBI method. In conclusion, the COMBI method is most effective when both screening approaches, i.e. SVM and filter screening, are applied simultaneously (**Supplementary Figure S4)**.

Besides checking whether easy simplifications of the COMBI method achieve the same effect, the performances of other competitor methods were investigated. As discussed in the **Discussion Section** of the main paper**,** there are a number of related machine learning methods, out of which we selected three as representatives to be compared to the COMBI method in this simulation setup.

The most related method separates the two steps of COMBI method, *i.e.* machine learning and statistical testing, from each other and was proposed by Wasserman and Roeder[11]. In the course of this algorithm, half of the data points (*i.e.* half of the individuals) are first randomly selected and an SVM is trained to identify the k SNPs with the highest corresponding weights. In the second step, p-values are computed on the other half of the data points considering only the SNPs identified in the previous step. Even though the significance threshold α can now simply be corrected with $\alpha_{new} = \alpha/k$ (which is much less conservative than the Bonferroni correction $\alpha_{Bonf.} = \alpha/d$ when considering all SNPs), this method comes with a loss of power (see **Supplementary Figure S13)**, where the corresponding curves are constantly below the curve of the regular COMBI method.

Meinshausen et al.[10] present an extension of the method proposed by Wasserman and Roeder[11]. Instead of splitting the data once into two sets and using one for SVM training and the other for statistical testing, they suggest aggregating the results of multiple random splits, arguing that this will decrease error rates and increase power. They propose using quantiles as summary statistics for the p-values of the multiple splits. After defining $q_\gamma(\cdot)$ to be the empirical γ-quantile function the p-value for each predictor j = 1, …, p is calculated with $P_j = \min\{1, (1 - \log \gamma_{min}) \inf_{\gamma \in (\gamma_{min}, 1)} Q_j(\gamma)\}$ where $Q_j(\gamma) = \min\{1, q_\gamma(\{P^{(b)}_j/\gamma\ ;\ b = 1, \ldots, B\})\}$. In our simulation, this method does not reach the performance level of the COMBI method (See **Supplementary Figure S13**) and also fails to reach that of the RPVT approach. The results indicate that it may be more effective to use the full data set for selection of candidate SNPs and multiple testing on these SNPs (as done in the course of the COMBI method), rather than using a subset for selection and another subset for testing (as done in the single- and multi-split methods by Wasserman and Roeder[11] and Meinshausen et al.[10]).

Another method for identifying associated regions was proposed by Roshan et al.[12]. It consists of a statistical testing step where the top χ^2-ranked SNPs are selected, and put into the next step to train an SVM. This basically boils down to a version of the COMBI method, where the order of the two steps (SVM training and statistical testing) is reversed. Applying this method to our 10,000 simulated datasets yields no gain in performance (See **Supplementary Figure S13**), suggesting that it is not of importance whether the SVM is trained on the whole dataset or on a subset of SNPs with high p-values. The ordering of the weights will remain the same for those top SNPs.

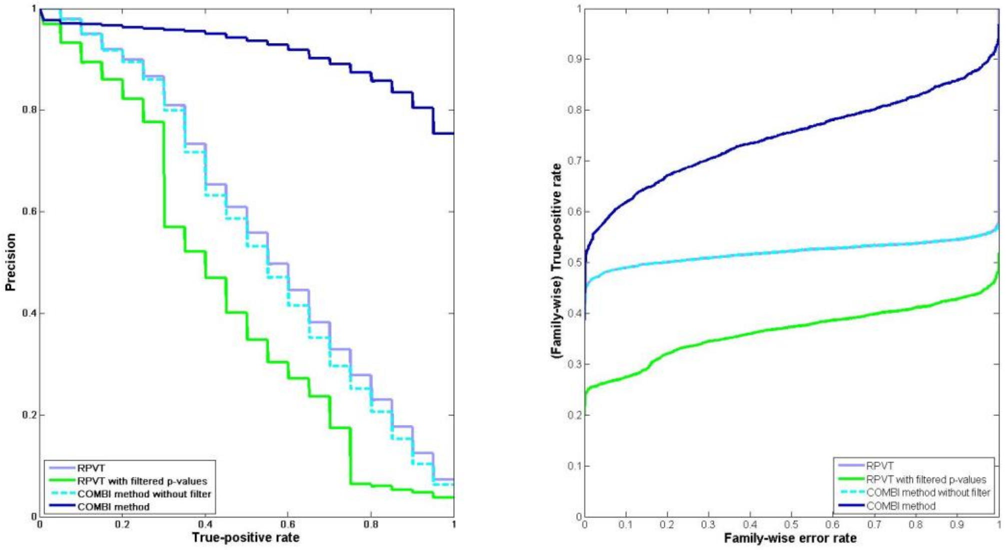

Supplementary Figure S12

Results of the simulation for various baseline algorithms presented via the precision-recall curve and the *FWER-TPR* curve. The COMBI method yields the most power only if the filtering step is applied (dark blue line); otherwise no effect in comparison to RPVT can be achieved (turquoise dashed and light blue line are almost identical). Applying a filter directly to the p-values does not have the same effect as the combined SVM and filter screening step (light green line). Both of these selection levels are therefore crucial. Separating the SVM selection and multiple testing step from each other yields a loss in power and cannot reach comparable effectiveness.

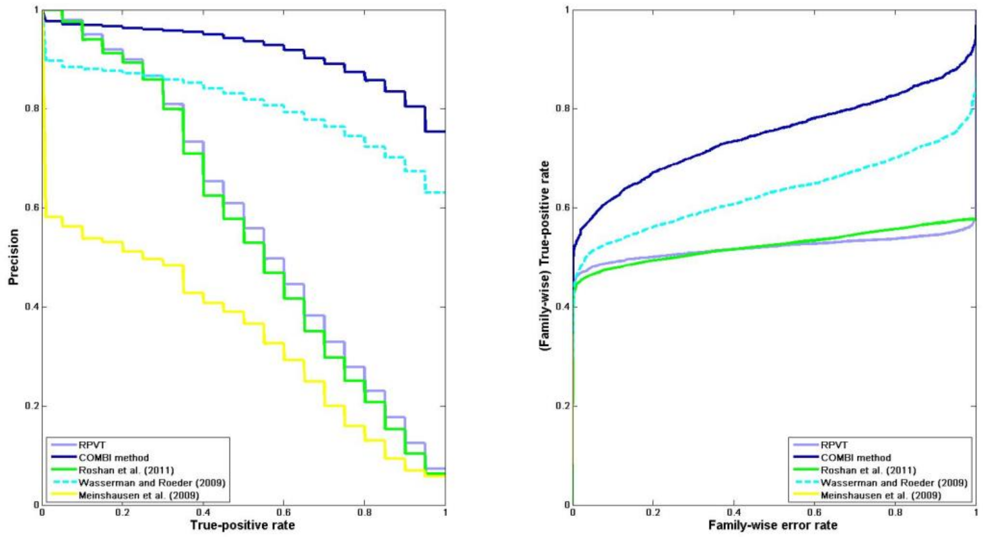

Supplementary Figure S13

Results of the simulation for various competitor algorithms presented via the precision-recall curve and the *FWER-TPR* curve. The COMBI method yields the highest power, while no other method can achieve the same performance. Note that the yellow curve is almost identical to the green one in the figure to the right, which is why it is hardly visible.

3. Analysis of real data (WTCCC, 2007)

3.1. Data preparation

Genotype and phenotype data for the seven disease and two control groups used in the WTCCC[1] were downloaded from the EGA website (European Genome-phenome Archive, ega.crg.eu) after being granted the corresponding access. Seven case-control datasets, one for each disease, were built combining both control groups. SNPs and samples that did not fulfill the quality control in the original WTCCC paper were removed from each dataset using the lists provided at the WTCCC site.

Based on lists provided by the WTCCC (see www.wtccc.org.uk), we removed an additional set of 579 false-positive SNPs from analysis (for instance, SNPs that are significant, isolated hits, with no significance in surrounding SNPs in high LD, i.e., with no "tower" around them). Since these lists seemed to lack the information corresponding to the coronary artery disease (CAD) study, all genome-wide significant SNPs (<5e-7) for that study that did not appear in the original WTCCC paper were manually removed.

3.2. Automatic validation procedure

Our automatic validation procedure is based on identifying associations to a disease reported in the GWAS catalog for either the target SNP or other SNPs in high LD with them. That is, to validate the SNPs COMBI identifies as good predictors upon analysis of the WTCCC data, we used the conservative criterion of ascertaining whether our candidate SNPs or other SNPs in high LD with them had been reported as associated to the corresponding disease by independent GWAS published after the WTCCC study. Our validation procedure considers a physical window of 200kb around any given SNP associated with a given disease and selects all SNPs within this window presenting strong LD with the target SNP. LD calculations were performed with PLINK[13] and were based on the genomic sequences of the 85 CEU individuals from Phase 1 of the 1000 Genomes Project. Runs with different thresholds were performed: (r^2>0.7, >0.8,

>0.9 and =1) without any change in our results. For the resulting high-LD SNPs, our algorithm queries the GWAS catalog to check if these SNPs have been associated to a disease with p-values <10^{-5}. A hit indicates that a GWAS other than the original WTCCC study reported this SNP to be associated with the relevant disease and, thus, that independent evidence validates the discovery.

3.3. Prediction performance

In **Supplementary Table S1**, we present the prediction performances of an SVM as used in the first step of the COMBI method applied to the WTCCC data in comparison to the reported performance rates of other machine learning methods presented in the **Discussion Section** of the main paper. We use the area under the receiver operating characteristic curve (AUC) as the measure of performance.

Supplementary Table S1

Reported Prediction Performances (AUC in %) of the SVM in the first step of the COMBI method and other machine learning methods.

	SVM (first step of COMBI method)	Mittag et al.[14]	Davies et al.[15]	Evans et al.[16]	Kooperberg et al.[17]	Wei et al.[18]
BD	70.4	61	-	66.8	-	-
CAD	62	-	60.2	60.0	-	-
CD	64.9	-	-	61.0	63.7	-
HT	65.8	-	-	62.7	-	-
RA	63	-	-	66.6	-	-
T1D	74.9	88	-	74.9	88	89
T2D	63.4	62	-	60.1	-	-

Note that the AUC values of the optimal parameter values and methodological settings are reported for all methods (*i.e.* we chose the most successful feature encoding, p-value feature

selection, statistical test, SVM solver etc., for the SVM of the COMBI method, and the optimal parameter values corresponding to the methodology applied in that paper for all other methods). The linear SVM of the COMBI method performs similarly well as most other competing methods, except for type 1 diabetes, where a non-linear kernel performs better. We excluded the results of Quevedo et al.[19] from this table, since they were flawed by a lack of a step in their data processing procedure (J.R. Quevedo, personal communication).

3.4. Stability analysis

Stability is a desirable property of any SNP-selection method: if a method is not stable, it could either indicate that too many locations are selected, meaning that the result contains a random subset of non-significant SNPs, or that not enough locations are selected, so that the result contains only a random subset of the significant SNPs.

To investigate stability, we proceeded as follows: the original data was randomly split into two equally sized subsets (of individuals), *A* and *B*, for a number of 10 repetitions. The method under scrutiny, i.e., either the COMBI method or standard RPVT, is applied separately to the data from sets *A* and *B*, leading to sets *S(A)* and *S(B)* of respectively reported SNPs. Using the Tanimoto Index[20] $T(S(A), S(B)) = \frac{|S(A) \cap S(B)|}{|S(A) \cup S(B)|}$, the similarity of these two sets and thus the stability of the used method were measured. Here $|S|$ denotes the cardinality of the set S. In this manner, the stability of the COMBI method can be compared to the stability of standard RPVT.

Simulation results considering internal stability of the two methods when applied to the WTCCC Crohn's Disease data are shown in **Supplementary Figure S14**. COMBI produces more stable results than RPVT. The Tanimoto stability index is plotted against the mean number of reported associations, *i.e.* $\frac{|S(A)|+|S(B)|}{2}$. When we repeatedly split the data into two parts and investigate how similar the results of the two methods are in the two subsets, we find that the

results of the COMBI method are more similar and thus more stable. This is true for all levels of *ENFR* and thus for the mean number of reported associations.

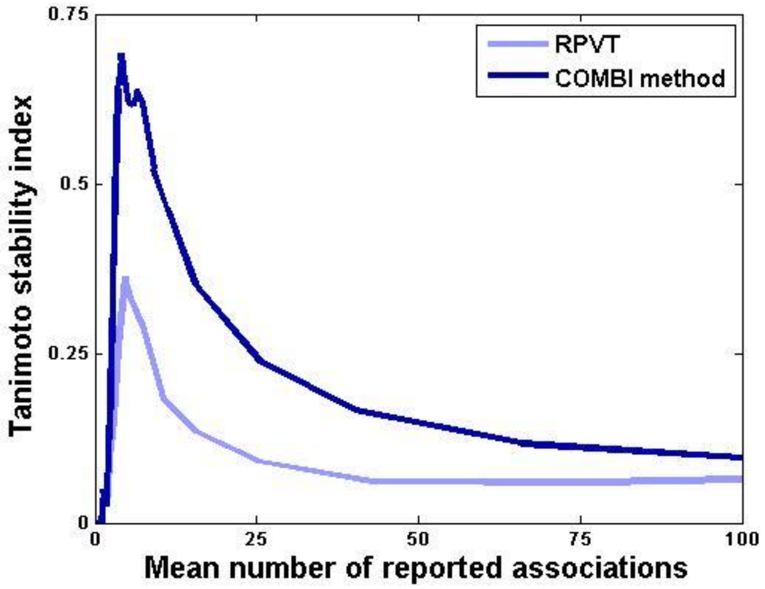

Supplementary Figure S14

Tanimoto Stability Indices for Crohn's Disease. We observe that the stability of the COMBI method is higher than that of RPVT. Note that higher Tanimoto index denotes higher stability.

This result holds for all seven diseases and is robust with respect to the choice of the parameter *k* (number of SNPs selected in the screening step) (See **Supplementary Fig. S15**).

Bipolar disorder **Coronary artery disease** **Hypertension**

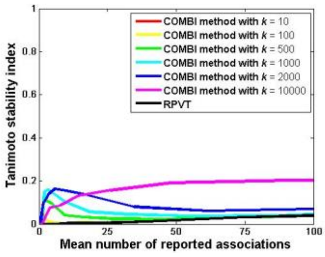

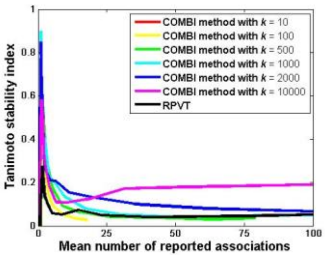

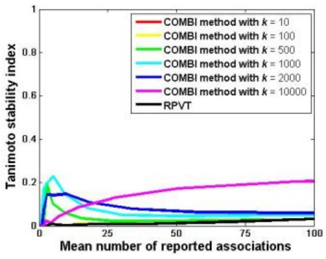

Rheumatoid arthritis **Type 1 diabetes** **Type 2 diabetes**

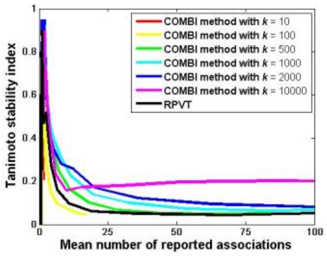

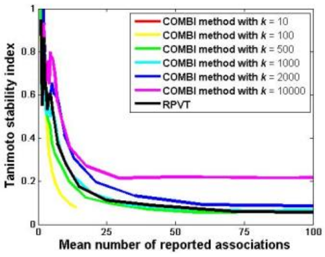

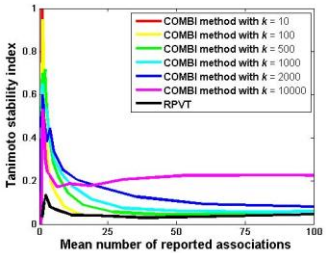

Supplementary Figure S15

Tanimoto Stability Indices for the other diseases. We observe that the stability is relatively robust to the choice of the parameter *k* (the number of SNPs to select in the screening step) of the COMBI method. Note that higher Tanimoto index denotes higher stability.

3.5. Functional study of two non-replicated SNPs

The automatic validation procedure that we used to ascertain whether SNPs COMBI detected using the original WTCCC data have been discovered and/or replicated by subsequent studies (described in **Supplementary Section 3.2.**) failed to validate two of the 46 significant associations COMBI detected. To evaluate the potential biological meaning of these two SNPs, we carried out a functional analysis. Results are reported in **Supplementary Table S2** below. See main text for discussion.

Supplementary Table S2

Functional analysis of two SNPs detected by COMBI and not replicated in subsequent GWAS studies.

	SNP detected by COMBI	
	**rs11110912**	**rs6950410**
**Disease**	HT (Hypertension)	T1D (Type 1 diabetes)
**Chr / Position (hg19)**	12:102042213	7:4038917
**Functional consequence**	Intronic MYBPC1 (myosin binding protein C)	Intronic SDK1 (sidekick cell adhesion molecule 1)
**OMIM[1]**	Yes (involved in familial hypertrophic cardiomyopathy)	-
**GWAS Catalog[2]**	No	No
**Genes (in 200 Kb window)**	MYBPC1, CHPT1, SYCP3	SDK1
**eQTL activity3 (P-value)**	CHPT1 (P<10-8)	-
**RegulomeDB[4]**	1d ("strong")	-
**Haploreg[5]**	Transcription factor activity *(BATF,PU1)*	DNAse activity (in Osteoblasts)

[1] OMIM. Role in disease evidence of the gene the associated SNP lies in (available at http://omim.org/)
[2] GWAS Catalog. Presence in the "reported gene" field in the GWAS Catalog (http://www.genome.gov/gwastudies/)
[3] eQTL activity. Evidence about the activity as eQTL in blood of the associated SNP (gathered from the "Blood eQTL browser"; http://genenetwork.nl/bloodeqtlbrowser/)
[4] RegulomeDB. Summary of DNA regulatory evidence (in http://regulomedb.org/)
[5] Haploreg. Noncoding regulatory evidence of the haplotype block (www.broadinstitute.org/mammals/haploreg/haploreg.php)

3.6. Comparison to Fast-LMM models

We compared COMBI against another state-of-the-art method by Lippert *et al.*[21] who devised a novel univariate analysis method to improve WTCCC findings and implemented a linear mixed model (LMM) to uncover new epistatic associations by means of brute force comparison of pairwise interactions. They applied both methods to the seven WTCCC diseases, searching for new univariate signals and for epistatic associations.

For the univariate analysis, they reported a total of 573 novel SNP-disease associations[21] with p-values less than $5x10^{-7}$), covering all WTCCC diseases but CAD, for which no novelty was reported. Most novel SNPs were mapped in the same loci, so we selected representative markers for each locus through the LD pruning option in PLINK. We computed pairwise LD with a sliding window of two SNPs (with steps of 1 SNP at a time). We discarded one SNP out each pair if they were in high LD ($R^2 \geq 0.8$). The final SNP lists, consisting in 1 for BD, 0 for CAD, 19 for CD, 1 for HT, 3 for RA, 39 for T1D and 9 for T2D, was run through our validation

pipeline using the same parameters that we use for COMBI in the present work (physical distance to tag-SNP: < 200 kb. Linkage disequilibrium with tag-SNP: $R^2 \geq 0.8$). The number of “true positive” hits (that is, of discoveries that have been validated in the literature) was limited, with only five of them featured in GWAS published after the WTCCC hits (CD: 3 hits, RA: 1 and T2D 1 hit each). This figure is much smaller than for COMBI (See **Supplementary Table S3**).

Supplementary Table S3 Not only does COMBI give rise to more validated discoveries, but these discoveries cover the whole range of WTCCC diseases.

Supplementary Table S3

Comparison of COMBI against LMM univariate method. Only the three diseases indicated in table were considered, as these were the only ones which reported hits by LMM validated in further independent studies.

DISEASE	COMBI set of SNPs	COMBI hits	LMM univariate set of SNPs	LMM univariate hits	Binomial p-value
Crohn's Disease	11	8	19	3	$4.07x10^{-5}$
Rheumatoid Arthritis	3	1	3	1	*NS*
Type 2 Diabetes	8	3	9	1	0.049

The epistasis analyses by Lippert *et al.*,[21] consisted of brute force computing of all possible pairwise SNP associations for 6 diseases (~63 billion pairs; no hits reported for Crohn´s disease), and testing their epistatic interaction in disease risk. The authors reported a final list consisting in 707 pairs of SNPs with p-values lower than 7.9×10^{-13} for each phenotype analyzed in WTCCC data. All individual SNPs taking part in the reported significant interactions were checked against our validation pipeline. We are aware that this is only a partial validation, since epistasis is not the simple addition of separated SNP effects, which are those that are registered in the GWAS catalog, but some associations still could emerge.

Applying the same LD pruning method than for the univariate method, we ended up with the following number of SNPs per each disease: 2 for BD, 32 for CAD, 0 for CD, 2 for HT, 7 for RA, 13 for T1D and 2 for T2D. By running these markers through our validation pipeline, we found a single association for T1D, while for the other diseases no markers were found. A comparison against COMBI hits for type 1 diabetes can be seen in **Supplementary Table S4**.

Supplementary Table S4

Comparison of COMBI against LMM epistatic method. Only T1D was subject to comparison.

**DISEASE**	**COMBI set of SNPs**	**COMBI hits**	**LMM epistatic set of SNPs**	**LMM epistatic hits**	**Binomial p-value**
Type 1 Diabetes	9	6	9	1	$1.1x10^{-4}$

3.7. Runtime analysis and implementation details

The COMBI method is implemented in R, Matlab/Octave and Java as a part of the GWASpi toolbox 2.0 (**https://bitbucket.org/gwas_combi/gwaspi/**, login user name: **gwas_combi_guest**, password: **combi123**.). The complete method is available in all languages and there are no substantial differences in implementation. See Supplementary Table S5 for implementation details

Supplementary Table S5: Specialities and details about used packages for all implementations of the COMBI method.

**Language**	**Speciality**	**Used packages**
**Java**	• COMBI method fully implemented<br>• supports other algorithms within the GWASpi software<br>• desktop computer oriented	libLinear[22], libSVM[23], apache commons math[24]
**Matlab/Octave**	• COMBI method fully implemented<br>• cluster oriented	libLinear[22]
**R**	• COMBI method fully implemented	LiblineaR[25], qqman[26], data.table[27], gtools[28], snpStats[29]

The runtime of the method depends on a variety of factors such as available cluster memory, hardware resources and operating system. For this analysis we have run the method with the implementation on the following technical platform: 40 * Intel(R) Xeon(R) CPU E5-2650 v3 @ 2.30GHz 64bit, 128GB RAM, Ubuntu 14.04.4 LTS (GNU/Linux 3.13.0-79-generic x86_64). The analysis of WTCCC's data on Crohn's disease chromosome 18 (assuming calculations on more chromosomes can be computed in parallel if necessary) took 9h 15min 24s using the Matlab/Ovtave implemenation. See **Supplementary Table S6** for information on how the computation time spread across the various steps of the COMBI method and how it varied for the different implementations in Java, Matlab/Octave and R.

Supplementary Table S6: Runtime analysis of the COMBI method. The complete method was applied to WTCCC data from chromosome 18 investigating Crohn's Disease. 1000 replications were run in both permutation procedures. Please note, that the permutation procedures are still being improved for both Java and R implementations and running times are provisional.

	**Matlab/Octave** (Here GNU Octave version 3.8.1)	**Java** (Here SUN JDK version 1.8.0_77 64bit, Maven version 3.3.9)	**R** (Here R version 3.0.2 (2013-09-25), Frisbee Sailing)
**Training of SVM (first step of COMBI)**	21s	8min 30s	41min 55s
**Calculation of raw p-values (second step of COMBI)**	3s	25s	1min 46s
**Permutation procedures for the calculation of significance thresholds**	9h15min	8d 3h 30min	>1month
**Overall**	9h15min24s	8d 3h 38min 55s	>1month

We should point out that with respect to running time, COMBI cannot compete with traditional GWAS methods like raw p-value thresholding. However, as we stress in the paper, we believe that the contribution of the present work lies in providing a new method that considerably improves power and precision rather than in improving running time.

References of supplementary material

1. Wellcome, T., Case, T. & Consortium, C. Genome-wide association study of 14,000 cases of seven common diseases and 3,000 shared controls. *Nature* **447,** 661–78 (2007).

2. Westfall, P. & Young, S. *Examples and Methods for p-Value Adjustment*. (Wiley, 1993).

3. Fan, J. & Lv, J. Sure independence screening for ultrahigh dimensional feature space. *J. R. Stat. Soc. Ser. B Statistical Methodol.* **70,** 849–911 (2008).

4. Alexander, D. H. & Lange, K. Stability selection for genome-wide association. *Genet. Epidemiol.* **35,** 722–728 (2011).

5. Salanti, G. *et al*. Underlying genetic models of inheritance in established Type 2 diabetes Associations. *American Journal of Epidemiology* **170(5)**, 537-545 (2009).

6. Clarke , G. M., Anderson, C. A., Pettersson, F. H., Cardon, L. R., Morris, A. P., & Zondervan, K. T. Basic Statistical analysis in genetic case-control studies. *Nature Protocols* ***6*(2)**, 121–133 (2011).

7. Marigorta, U.M. & Navarro, A. High Trans-ethnic Replicability of GWAS Results Implies Common Causal Variants. *Plos Genetics*, **9(6)**, e1003566 (2013).

8. Marigorta, U.M., Rodriguez, J.A. & Navarro, A. "GWAS: a milestone in the road from genotypes to phenotypes" in Appasani, K. (ed.). Genome-Wide Association Studies: From Polymorphism to Personalized Medicine. *Cambridge University Press*, pp. 12–25. (2016).

9. Gibson, G. Rare and common variants: twenty arguments. *Nature Review Genetics* **13**, 135-145 (2012).

10. Meinshausen, N., Meier, L. & Bühlmann, P. p-Values for High-Dimensional Regression. *J. Am. Stat. Assoc.* **104,** 1671 (2009).

11. Wasserman, L. & Roeder, K. High-dimensional variable selection. *Ann. Stat.* 2178–2201. doi:10.1214/08-AOS646 (2009).

12. Roshan, U., Chikkagoudar, S., Wei, Z., Wang, K. & Hakonarson, H. Ranking causal variants and associated regions in genome-wide association studies by the support vector machine and random forest. *Nucleic Acids Res.* **39 ,** e62–e62 (2011).

13. Purcell, S. *et al.* PLINK: a tool set for whole-genome association and population-based linkage analyses. *Am j Hum. Genet* **81,** 559–575 (2007).

14. Mittag, F. *et al.* Use of support vector machines for disease risk prediction in genome-wide association studies: Concerns and opportunities. *Hum. Mutat.* **33,** 1708–1718 (2012).

15. Davies, R. W. *et al.* Improved Prediction of Cardiovascular Disease Based on a Panel of Single Nucleotide Polymorphisms Identified Through Genome-Wide Association Studies. *Circ. Cardiovasc. Genet.* **3 ,** 468–474 (2010).

16. Evans, D. M., Visscher, P. M. & Wray, N. R. Harnessing the information contained within genome-wide association studies to improve individual prediction of complex disease risk. *Hum. Mol. Genet.* **18,** 3525–31 (2009).

17. Kooperberg, C., LeBlanc, M. & Obenchain, V. Risk prediction using genome-wide association studies. *Genet. Epidemiol.* **34,** 643–52 (2010).

18. Wei, Z. *et al.* From Disease Association to Risk Assessment: An Optimistic View from Genome-Wide Association Studies on Type 1 Diabetes. *PLoS Genet* **5,** e1000678 (2009).

19. Quevedo, J. R., Bahamonde, A., Perez-Enciso, M. & Luaces, O. Disease Liability Prediction from Large Scale Genotyping Data Using Classifiers with a Reject Option. *IEEE/ACM Trans. Comput. Biol. Bioinforma.* **9,** 88–97 (2012).

20. Rogers, D. J. & Tanimoto, T. T. A Computer Program for Classifying Plants. *Sci.* **132,** 1115–1118 (1960)

21. Lippert, C., Listgarten, J., Davidson, R.I., Baxter, J., Poon, H., Kadi, C.M. & Heckerman, D. An exhaustive epistatic SNP association analysis on expanded Wellcome Trust data. *Sci. Rep.* **3,** 1099 (2013).

22. Fan, R.-E., Chang, K.-W., Hsieh, C.-J., Wang, X.-R. & Lin, C.-J. LIBLINEAR: A Library for Large Linear Classification. *Journal of Machine Learning Research* **9**, 1871-1874. Software available at http://www.csie.ntu.edu.tw/~cjlin/liblinear (2008).

23. Chang, C.-C. & Lin, C.-L. LIBSVM: a library for support vector machines. *ACM Transactions on Intelligent Systems and Technology* **2(27)**, 1-27. Software available at http://www.csie.ntu.edu.tw/~cjlin/libsvm (2011).

24. The Apache Software Foundation. Commons Math: The Apache Commons Mathematics Library. Java version 1.7. Software available at http://commons.apache.org/proper/commons-math/ (2016).

25. Helleputte, T. & Gramme, P. LiblineaR: Linear Predictive Models Based on the LIBLINEAR C/C++ Library. R package version 1.94-2 from http://dnalytics.com/liblinear/ (2015).

26. Turner, S.D. qqman: an R package for visualizing GWAS results using Q-Q and manhattan plots. biorXiv DOI: 10.1101/005165. R package version 0.1.2 from http://cran.r-project.org/web/packages/qqman/ (2014).

27. Dowle, M., Srinivasan, A., Short, T. & Lianoglou, S. with contributions from Saporta, R. & Antonyan, E. data.table: Extension of Data.frame. R package version 1.9.6. from https://CRAN.R-project.org/package=data.table (2015).

28. Warnes, G.R., Bolker, B. & Lumley, T. gtools: Various R Programming Tools. R package version 3.5.0. from https://CRAN.R-project.org/package=gtools (2015).

29. Clayton, D. *snpStats: SnpMatrix and XSnpMatrix classes and methods*. R package version 1.22.0 from http://bioconductor.org/packages/release/bioc/html/snpStats.html (2015).
